# Fucoxanthin: A Promising Phytochemical on Diverse Pharmacological Targets

**DOI:** 10.3389/fphar.2022.929442

**Published:** 2022-08-02

**Authors:** Mumtaza Mumu, Ayan Das, Talha Bin Emran, Saikat Mitra, Fahadul Islam, Arpita Roy, Md. Mobarak Karim, Rajib Das, Moon Nyeo Park, Deepak Chandran, Rohit Sharma, Mayeen Uddin Khandaker, Abubakr M. Idris, Bonglee Kim

**Affiliations:** ^1^ Department of Biochemistry and Molecular Biology, Faculty of Biological Sciences, University of Chittagong, Chittagong, Bangladesh; ^2^ Department of Pharmacy, BGC Trust University Bangladesh, Chittagong, Bangladesh; ^3^ Department of Pharmacy, Faculty of Allied Health Sciences, Daffodil International University, Dhaka, Bangladesh; ^4^ Department of Pharmacy, Faculty of Pharmacy, University of Dhaka, Dhaka, Bangladesh; ^5^ Department of Biotechnology, School of Engineering and Technology, Sharda University, Greater Noida, India; ^6^ Department of Biomedical Engineering, University of Houston, Houston, TX, United States; ^7^ Department of Pathology College of Korean Medicine, Kyung Hee University, Seoul, South Korea; ^8^ Department of Veterinary Sciences and Animal Husbandry, Amrita School of Agricultural Sciences, Amrita Vishwa Vidyapeetham University, Coimbatore, India; ^9^ Department of Rasa Shastra and Bhaishajya Kalpana, Faculty of Ayurveda, Institute of Medical Sciences, Banaras Hindu University, Varanasi, India; ^10^ Centre for Applied Physics and Radiation Technologies, School of Engineering and Technology, Sunway University, Bandar Sunway, Malaysia; ^11^ Department of Chemistry, College of Science, King Khalid University, Abha, Saudi Arabia; ^12^ Research Center for Advanced Materials Science (RCAMS), King Khalid University, Abha, Saudi Arabia

**Keywords:** fucoxanthin, phytochemicals, antimicrobial activity, anticancer activity, anti-inflammatory activity, enzyme inhibition

## Abstract

Fucoxanthin (FX) is a special carotenoid having an allenic bond in its structure. FX is extracted from a variety of algae and edible seaweeds. It has been proved to contain numerous health benefits and preventive effects against diseases like diabetes, obesity, liver cirrhosis, malignant cancer, etc. Thus, FX can be used as a potent source of both pharmacological and nutritional ingredient to prevent infectious diseases. In this review, we gathered the information regarding the current findings on antimicrobial, antioxidant, anti-inflammatory, skin protective, anti-obesity, antidiabetic, hepatoprotective, and other properties of FX including its bioavailability and stability characteristics. This review aims to assist further biochemical studies in order to develop further pharmaceutical assets and nutritional products in combination with FX and its various metabolites.

## Introduction

Nutrition plays a major role in preventing lifestyle diseases such as obesity, heart diseases, cancer, diabetes, and other long-term diseases (Esselstyn, 2007; Sirtori et al., 2009). Hence, there has been a growing interest in detecting functional and non-toxic elements from foods to treat and maintain different physical illnesses. FX, a marine carotenoid, is one of these effective ingredients. It is about >10% of the approximated total naturally occurring carotenoids (Peng et al., 2011). FX is an orange pigment, present in chloroplasts of macroalgae and microalgae as a major carotenoid (Moreau et al., 2006; Afolayan et al., 2008; Miyata et al., 2009; Mise et al., 2011; Kim SM. et al., 2012; D’Orazio et al., 2012; Dai et al., 2021). R. Willstatter and H. J. Page (Aronoff, 1950) first extracted FX from three marine seaweeds, *Fucus*, *Dictyota*, and *Laminaria*, in 1914 and Englert *et al.* (1990) determined the complete chiral structure of FX. The remarkable pharmacological activities of FX are related to its unique molecular structure. Chemically, FX has a molecular formula of C_42_H_58_O_6_ with a molecular weight of 658.906 g/mol. Its structure is similar to neoxanthin, dinoxanthin, and peridinin and different from other carotenoids like β-carotene and astaxanthin. FX contains an unusual allenic bond, a 5,6-monoepoxide, and nine conjugated double bonds, and some oxygenic functional groups including hydroxyl, epoxy, carbonyl, and carboxyl moieties (Hosokawa et al., 2009; Gundermann and Büchel, 2014). Until now, 43 of the total approximately 700 naturally occurring carotenoids have been found to contain the allene group and in brown seaweeds, and the first isolated allenic carotenoid was FX (Dembitsky and Maoka, 2007; Britton et al., 2008). FX is more functional because of this exceptional allenic bond (Dembitsky and Maoka, 2007). The water solubility of FX is 0.00057 g/L as determined by the ALOGPS tool (Tetko et al., 2005). Heat, light, oxygen, enzymes, unsaturated lipids, and other prooxidant molecules can easily affect the structure and chirality of FX and make it unstable (Zhao et al., 2014). FX breakdown is initiated at 25–60°C temperature in the absence of light and air at pH 4.6. pH 7.4 can diminish the degradation of FX (Zhao et al., 2019). All carotenoids exist in two isomeric configurations, trans or cis, because of the presence of conjugated double bonds in their polyene chains. Although the major isomer of FX is all-trans FX, the increase of extraction temperature can increase the ratio of cis FX (Kawee-Ai et al., 2013; Susanto et al., 2017). Additionally, the cis-form of FX can be enhanced by ultrasonic therapy. Cis FX shows less antioxidant activity than all-trans FX (Nakazawa et al., 2009; Kawee-Ai et al., 2013).

Generally, microalgae have a higher concentration of FX than macroalgae (Kim S. M. et al., 2012). *Chlorophyceae* (Green algae), *Rhodophyceae* (Red algae), and *Phaeophyceae* (Brown algae) are the three major classes of marine algae (Khan et al., 2010). Among them, *Phaeophyceae* usually contain FX, which absorbs blue and green lights and gives them a brownish-olive color. They are mostly found in both cold and temperate oceans worldwide. The sources of FX and its distribution are represented in Table 1. Few brown seaweeds containing FX are edible macroalgae in Southeast Asia and certain European nations. Among them, *Undaria* (known as Wakame), *Laminaria* (known as Konbu), and *Hijikia* (known as Hijiki) are the most common seaweeds as a source of food (MacArtain et al., 2007).

**TABLE 1:** Sources of FX and their distribution.

Microalgae/Macroalgae	Name	Distribution	Ref
Brown seaweeds (Macroalgae)	*Alaria crassifolia*	Japan	Airanthi et al. (2011a)
	*Ascophyllum nodosum*	Europe, East Greenland, North America	Stengel and Dring, (1998)
	*Cladosiphon okamuranus*	Japan	Mise et al. (2011)
	*Cystoseira hakodatensis*	Mediterranean, Indian, and Pacific Oceans	Airanthi et al. (2011a), Airanthi et al. (2011b)
	*Ecklonia stolonifera*	Japan	Jung et al. (2016)
	*Eisenia bicyclis*	Pacific Ocean waters centered near Japan, South Korea	Airanthi et al. (2011a)
	*Fucus serratus*	Atlantic coast of Europe from Svalbard to Portugal, Canary Islands, North-east America Iceland, and Faroe Island	Strand et al. (1998)
	*Fucus vesiculosus*	Europe, Northern Russia, the Baltic Sea, Greenland, Azores, Canary Islands, Morocco, Madeira, Atlantic coast of North America from Ellesmere, Hudson Bay to North Carolina	Zaragozá et al. (2008)
	*Hijikia fusiformis*	Japan, Korea, and China	Nishino, (1998), Yan et al. (1999)
	*Himanthalia elongata*	The Baltic Sea, the North Sea, the north east Atlantic Sea	Rajauria et al. (2017)
	*Ishige okamurae*	Japan	Kil-Nam Kim et al. (2010)
	*Kjellmaniella crassifolia*	Japan	Airanthi et al. (2011a)
	Laminaria digitata	Greenland, Russia, Iceland, France, England	Haugan and Liaaen-Jensen (1992), Shannon and Abu-Ghannam, (2017)
	*Laminaria japonica*	China, Japan and Korea	Das et al. (2008), Zhang et al. (2008), Miyata et al. (2009)
	*Laminaria ochotensis*	China, Japan and Korea	Miyata et al. (2009)
	*Laminaria religiosa*	Japan	Mori et al. (2004)
	*Myagropsis myagroides*	Japan	Heo et al. (2010)
	*Padina tetrastromatica*	Somalia	Sangeetha et al. (2010)
	*Petalonia binghamiae*	California	Murakami et al. (2002)
	*Sargassum fulvellum*	Coasts of the eastern and southern sea of Korea	Miyata et al. (2009)
	*Sargassum heterophyllum*	South Africa	Afolayan et al. (2008)
	*Sargassum horneri*	Japan and Korea	Airanthi et al. (2011b)
	*Sargassum siliquastrum*	Japan	Heo et al. (2008)
	Scytosiphon lomentaria	Denmark	Mori et al. (2004)
	*Undaria pinnatifida*	Japan, Korea, China, Europe, North America, South America and Australasia	Yan et al. (1999), Hosokawa et al. (1999), Ikeda et al. (2003), Hosokawa et al. (2004), Maeda et al. (2005), Sachindra et al. (2007), Asai et al. (2008), Khan et al. (2008b), Liu et al. (2009), Nakazawa et al. (2009), Airanthi et al. (2011b), Okada et al. (2011)
Diatoms (Microalgae)	*Chaetoseros sp.*	North America	Iio et al. (2011a), Iio et al. (2011b)
	*Cylindrotheca Closterium*	Northern European seas	Rijstenbil, (2003)
	*Isochrysis galbana*	Coastal; Atlantic	Raposo et al. (2015), Delbrut et al. (2018)
	*Nitzschia laevis*	Germany, Argentina	Bingbing Guo et al. (2020)
	*Odontella aurita*	Denmark, Argentina, Brazil, and Paraguay	Moreau et al. (2006)
	*Phaeodactylum tricornutum*	Both brackish and marine waters worldwide	Nomura et al. (1997)
	*Tisochrysis lutea*	Worldwide	Mohamadnia et al. (2020)
	*Turbinaria triquetra*	Tropical marine waters	Mohamadnia et al. (2020)

In algae, FX forms a light-harvesting system with chlorophyll and significantly contributes to photosynthesis by absorbing light energy as well as protects chlorophyll from the harmful photooxidative effects of bright light (Takaichi, 2011). It transfers more than 60% of the energy to chlorophyll-a in diatoms (Streckaite et al., 2018). FX also establishes biological functions in marine animals. The enhanced immunological activity and ovulation in sea urchins are one of them (Tsushima, 2007; Yang et al., 2021). Humans can easily absorb FX because of its small molecular weight. Additionally, it is nontoxic and exerts several beneficial biological activities in the human body. Like other carotenoids, FX is a potent antioxidant due to its singlet oxygen-quenching functionality (Sachindra et al., 2007; Galasso et al., 2017). It has also received more attention as an anticancer carotenoid. It comprises other numerous pharmacological activities, namely, anti-obesity, anti-inflammatory, antibacterial, antimalarial, antidiabetic, neuroprotective, liver-protective, anti-osteoporosis activities, regulation of intestinal flora, etc. (Krinsky et al., 2004; Kotake-Nara et al., 2005b; Gammone and D'Orazio, 2015; Zorofchian Moghadamtousi et al., 2014). In recent years, FX has been widely investigated for uses in foods, nutraceutical pharmaceuticals, and cosmeceutical industries. Although FX can be produced by chemical synthesis, edible oil extraction from algae is more accessible, safe, and utilizes as FX containing beneficial food without any additional purifications (Miyashita et al., 2020). Industrially, diatoms are the most useful sources because they contain four times more FX than brown seaweeds (Wang et al., 2018). Among all diatoms, *Tisochrysis lutea* provides a higher concentration of FX, and because it does not have a cell wall, it is easy to extract FX from this diatom (Delbrut et al., 2018; Gao et al., 2020; Mohamadnia et al., 2020; Mohamadnia et al., 2021).

Therefore, this article summarizes the present obtainable scientific literatures concerning the metabolism, bioavailability, and health benefits of FX such as its antimicrobial, anti-plasmodial, antioxidant, anti-inflammatory, antiallergic, and anti-obesity effects as well as obesity-related disease prevention including antidiabetic, anti-cardiovascular properties, tumor inhibition, and its protective roles on bones, skin, eyes, and liver.

## Fucoxanthin Metabolism

The metabolic fates of FX in humans and other animals indicate that fucoxanthinol (FXol), amarouciaxanthin A, amarouciaxanthin B, halocynthiaxanthin, mytiloxanthin, crasssostreaxanthin A, FXol-3′-sulfate, etc., are some main metabolites of FX. Several investigations were conducted to characterize the metabolic pathway of FX in mice. After oral administration, FX is rapidly deacetylated to FXol by lipase and esterase in the gastrointestinal tract of mice within 2 h. The dietary FX was absorbed in the intestine as FXol, and no pure FX has been found in the plasma or liver tissue of mice (World Health Organization, 1981). In HepG2 cells and in the liver microsomes of ICR mice, further conversion of FXol produced amarouciaxanthin A through dehydrogenation/isomerization (Asai et al., 2004). Amarouciaxanthin A is a marine carotenoid, first found in *Amaroucium pliciferum* (a tunicate) (Matsuno et al., 1985)*.*

FX metabolism is quite different in humans and mice (Hashimoto et al., 2012). An *in vitro* study by Sugawara *et al.* (Sugawara et al., 2002) showed that FX was absorbed as FXol in differentiated Caco-2 cells. The Caco-2 cell is an excellent model for determining the metabolism and absorption of a compound by human intestinal cells (Angelis and Turco, 2011; Hidalgo et al., 2011). Generally, no fatty acid esters of carotenoids are found in human chylomicrons or serum, which also indicates that esters are hydrolyzed in the human small intestine (Khachik et al., 1992; Wingerath et al., 1995; Chitchumroonchokchai and Failla, 2006; Schilter et al., 2010; Wang et al., 2022). Hence, like other carotenoids, dietary FX might be deacetylated by lipase and carboxylesterase in the intestinal tract of humans. NAD^+^ works as a cofactor in this process. As no amarouciaxanthin A was founded in plasma, FXol is seemed to be the primary active metabolite in humans. Further biotransformation of FXol to unknown metabolites might be taking place in the human body (Peng et al., 2011; Viera et al., 2018).

## Bioavailability of FX

Metabolism, absorption, bioaccessibility, transport, and tissue distribution are some factors that are closely related to the concept of bioavailability (Fernández-García et al., 2012). Although FX provides promising bioactivities and pharmacological effects, its wider applications in food, cosmetics, and drug industries are limited because of its weak stability (Hii et al., 2010; Zhao et al., 2014; Zhao et al., 2019) and relatively lower bioavailability (Hashimoto et al., 2012). Many researches demonstrated that very few carotenoids like α-carotene and β-carotene are generally consumed in the human small intestine and then transformed into vitamin A (Poor et al., 1993; Oshima et al., 1999; Schweigert et al., 2001). FX is an epoxy-xanthophylls and non-provitamin A-type carotenoid. Although studies reported the metabolism and accumulation of some xanthophylls such as astaxanthin and canthaxanthin (World Health Organization, 1981; Odeberg et al., 2003; During and Harrison, 2004; Showalter et al., 2004; Petri and Lundebye, 2007; Yonekura and Nagao, 2007; Viera et al., 2018), non-provitamin A-type carotenoids (like FX) have insufficient metabolism and bio-accessible information to describe their bioavailability.

The bioavailability of FXol is higher than that of FX; it is lower than other nutritional carotenoids like lutein, β-carotene, and astaxanthin (Viera et al., 2018). There are many hypotheses that might explain the FX’s low bioavailability, including the presence of dietary fiber in the algal matrix (Yonekura and Nagao, 2009), a faster first-pass metabolic activity after the intestinal uptake by detoxification enzymes (Asai et al., 2008), or a less affinity of the intestinal transporters because of a lower level of lipophilicity of FX than its metabolic properties (Asai et al., 2008). Besides, the lipophilicity of FX and its metabolites do not influence their tissue distribution, but their higher lipophilicity can decrease depletion (Hashimoto et al., 2012). FX contains a polyunsaturated structure and thus heat and acidic environment can affect its stability (Zhao et al., 2014). Additionally, the degradation of FX can be increased by oxygen, light, and heavy metal exposure during the processing and storage (Pinto et al., 2003; Hii et al., 2010; Sun et al., 2018). Zhao *et al.* (Zhao et al., 2019) reported that pH is the most influential factor on FX stability, followed by temperature and light. According to their results, increasing the temperature from 25 to 60°C substantially increased the FX breakdown. Lowering the pH to 1.2 also mediated FX breakdown, while the degradation was retarded by raising the pH to 7.4 (Zhao et al., 2019).

Because of the low bioaccessibility of FX in humans, different approaches have been developed to increase its absorption, efficiency, and stability. Fish oil and medium-chain triacylglycerols (MCTs) can increase the absorption rate of FX as it is highly soluble in these media (Maeda et al., 2007b; Sachindra et al., 2007). FX, oleic acid (OA), and bovine serum albumin (BSA) can form complexes that can improve the intestinal absorption of FX in water (Liu Y. et al., 2019). Again, the low bioaccessibility of FX can be improved by using encapsulated FX. FX can be encapsulated with biopolymers like maltodextrin (MD), gum arabic (GA), and whey protein isolate (WPI), which improves the thermal stability of FX and intestinal absorption (Sun et al., 2018). Caseinate-stabilized zein particle (zein-Cas)-encapsulated FX also shows efficiently higher bioaccessibility than non-encapsulated FX (Bray et al., 2018). Its dispersibility and bioaccessibility can be enhanced by complete encapsulation within the hydrophilic OA–BSA (oleic acid and bovine serum albumin) particles (Li et al., 2021). So, natural emulsion-based delivery systems with different types of emulsifiers can develop the formation, stability, and bioaccessibility of FX (Ma et al., 2019).

## FX as a Multi-Functional Nutrient in Health Benefits

### Antimicrobial Activity

Microbes can easily adapt to new environments, resist old drug treatments, and have a high spreading rate. Nowadays, drug resistance has become a challenging issue for the whole world. For this reason, knowledge concerning microbes has been incredibly increased during the 20th century. Thus, scientists are trying to find newer effective natural antimicrobial agents from bioactive compounds with better potential efficacy against microbes, fewer side effects, better bioavailability than older antibiotics, and minimum toxicity. Carotenoids, especially FX, contain remarkable antimicrobial properties. Thus, FX can be used as a potential inhibitor to prevent various microbial diseases.

### Antibacterial Activity

The brown macroalgae extracts inhibit the growth of pathogens more efficiently than the green macroalgae (Lavanya and Veerappan, 2011). FX, the main carotenoid of marine brown seaweed and diatoms, has been reported to show antibiotic properties (Pérez et al., 2016; Shannon and Abu-Ghannam, 2016). FX extracted from *Himanthalia elongate* (Irish brown seaweed) was tested for its potential antimicrobial behaviors against *Listeria monocytogenes* bacteria. The zone of growth inhibition (ZOI) for *Listeria monocytogenes* was 10.89 mm (Rajauria and Abu-Ghannam, 2013). Abou (Abou, 2013) investigated the antibacterial activities of some seaweeds. They extracted FX from *Turbinaria triquetra*, which was recorded to have the highest antibacterial effect against all the selected bacteria including *Staphylococcus aureus*, *Escherichia coli*, *Bacillus subtilis*, *Bacillus cereus*, *Klebsiella pneumoniae*, and *Pseudomonas aeruginosa.* The highest activity of FX (100 µgml^−1^) was against *Bacillus subtilis* (ZOI 7.0 mm) (Abou, 2013). Similarly, Karpiński and Adamczak (Karpiński and Adamczak, 2019) observed the antibacterial activities of FX against 20 bacterial species. They found that the mean ZOIs varied from 9.0 to 12.2 mm for Gram-positive bacteria and 7.2–10.2 mm for Gram-negative bacteria. The highest diameter of the inhibition zone was 12.2 mm at a concentration of 62.5 µgml^−1^ FX against *Streptococcus agalactiae.* Zonglin Liu Z. et al. (2019) reported the antibacterial properties of FX isolated from *Undaria pinnatifida* (an edible seaweed) against five human pathogens. According to their *in vitro* study, FX effectively suppressed the growth of *Staphylococcus aureus*, *Bacillus subtilis*, *Enterococcus faecalis*, *Enterococcus sp.,* and *Pseudomonas aeruginosa*. Likewise, several studies have reported that FX shows lower antibacterial effects against Gram-negative bacteria than Gram-positive bacteria (Abou, 2013; Karpiński and Adamczak, 2019). This selective antibacterial activity may be influenced by many factors such as charging densities, the lipopolysaccharide (LPS) structure, and different cytoplasmic membrane lipid compositions in Gram-negative and Gram-positive bacteria (Devine and Hancock, 2002). Generally, when an infection is caused by Gram-negative bacteria, FX can help to reduce inflammation. Conversely, FX is ineffective against strict anaerobic bacteria (Karpiński and Adamczak, 2019). The antibacterial activities of FX along with their bacterial inhibition zones are summarized in Table 2.

**TABLE 2:** Antibacterial and antifungal activities of FX-containing algae.

Pharmacological effects	Algae species containing FX	Inhibitory activities	Ref
*Antibacterial activity*	*Himanthalia elongate*	Inhibits the growth of *Listeria monocytogenes* bacteria	Rajauria and Abu-Ghannam, (2013)
	*Turbinaria triquetra*	Inhibits the growth of *Staphylococcus aureus*, *Escherichia coli*, *Bacillus subtilis*, *Bacillus cereus*, *Klebsiella pneumoniae*, and *Pseudomonas aeruginosa*	Abou, (2013)
	*Undaria pinnatifida*	Inhibits the growth of five human pathogens (*Staphylococcus aureus*, *Bacillus subtilis*, *Enterococcus faecalis*, *Enterococcus sp.,* and *Pseudomonas aeruginosa)*	Zonglin Liu et al. (2019)
	*Saccharina japonica, Sargassum horneri*	Inhibits the growth of *Staphylococcus aureus, Bacillus cereus, Listeria monocytogenes, Escherichia coli*	Sivagnanam et al. (2015)
	*Pavlova lutheri, Isochrysis galbana, Navicula sp., Chaetoceros calcitrans, Dunaliella salina, Thalassiosira sp., Chaetoceros gracilis*	Inhibits the growth of *Bacillus subtilis, Staphylococcus aureus, Escherichia coli, Klebsiella pneumoniae, Pseudomonas aeruginosa*	Peraman and Nachimuthu (2019)
*Antifungal activity*	*Saccharina japonica, Sargassum horneri*	Inhibits the growth of *Candida albicans, Aspergillus brasiliensis*	Sivagnanam et al. (2015)
	*Pavlova lutheri, Isochrysis galbana, Navicula sp., Chaetoceros calcitrans, Dunaliella salina, Thalassiosira sp., Chaetoceros gracilis*	Inhibits the growth of *Aspergillus brasiliensis, Aspergillus fumigatus, Candida albicans*	Peraman and Nachimuthu, (2019)

### Antifungal and Antiviral Activities

Despite the fact that FX has considerable properties to be as an antifungal agent, there are just a few studies that have investigated it. An *in vitro* study reported that different oil extracts from *Sargassum horneri* and *Saccharina japonica*, which contain FX, exhibited excellent antifungal properties against *Aspergillus brasiliensis* and *Candida albicans*. According to their results, the acetone–methanol mix extract of *Sargassum horneri* shows the highest antimicrobial activity against both fungi (Sivagnanam et al., 2015). Additionally, in a recent *in vitro* study, seven FX containing microalgae (*Pavlova lutheri*, *Isochrysis galbana*, *Navicula sp.*, *Chaetoceros calcitrans*, *Dunaliella salina*, *Thalassiosira sp.,* and *Chaetoceros gracilis*) were screened for their antifungal activities against *Aspergillus fumigatus*, *Aspergillus brasiliensis*, and *Candida albicans*. At MIC:40 mg/ml, *Dunaliella salina* showed an effective antifungal activity against all the three fungi, with the percentage of growth inhibition of 89.26, 87.67, and 81.02 for *Aspergillus brasiliensis*, *Aspergillus fumigatus,* and *Candida albicans,* respectively. Notably, *Chaetoceros gracilis* extracts also showed excellent inhibitory activity (Peraman and Nachimuthu, 2019). Table 2 represents the antifungal properties along with the antibacterial activities of FX as well as their inhibition zones.

Additionally, antiviral activities of FX have been appraised in a plenty of experimental studies. An *in vitro* study utilizing Raji cells estimated that at a lower concentration, FX and its metabolites could inhibit 12-O-tetradecanoylphorbol-13-acetate (TPA)-mediated Epstein–Barr virus activation. Among all the metabolites, halocynthiaxanthin (a metabolite of FX in marine animals) shows the strongest inhibitory activity at 500 and 100 M ratios. They showed cytotoxicity at a higher concentration (1000 and/or 500 M ratio per TPA) (Tsushima et al., 1995). Several FX containing seaweeds might be able to reduce the degree of angiotensin-converting enzyme (ACE)/angiotensin II (AngII)/angiotensin receptor II type 1 (ATR1) axis dominance by inhibiting ACE in COVID-19 patients (Tamama, 2020). But the role of FX in this process is still unknown.

### Antimalarial and Anthelmintic Activities

Malarial disease is transmitted by mosquitoes and affects humans and other animals (Mordecai et al., 2013; Cowman et al., 2016). It is caused by *Plasmodium* microorganisms and single-celled microorganisms. Among them, *Plasmodium falciparum* is the most common cause of malarial death (Snow et al., 2005; Noor et al., 2014). Even though natural products such as quinine and artemisinin, as well as their synthetic derivatives, have been the backbone of antimalarial chemotherapy for the last 100 years, resistance to these medicines has created the requirement of new treatments to treat the corresponding diseases (Sidhu et al., 2006; Tyagi et al., 2018). Marine compounds can be used to prevent malaria because of their potential biological activities. Afolayan et al. (2008) investigated the antiplasmodial activity of four compounds, sargaquinoic acid, sargaquinal, sargahydroquinoic acid, and FX, which were extracted from the South African algae *Sargassum heterophyllum*. Their results exhibited that FX had the highest antimalarial effect (IC_50_ = 1.5 μg ml^−1^) against a chloroquine-sensitive strain (D10) of *Plasmodium falciparum.* Although the antiplasmodial effects of FX may be linked to their antioxidant capabilities, further study is required to identify their mechanism of this action (Afolayan et al., 2008). Peraman and Nachimuthu investigated the anthelmintic activity of FX containing seven microalgae at 20, 40, and 80 mg/ml concentrations against *P. posthuma* (test earthworms). Based on their data, *Isochrysis galbana* and *Chaetoceros gracilis*, containing the highest amount of FX (5.93 and 1.92 mg/g, respectively), exhibited a greater potential anthelmintic activity. Both *Isochrysis galbana* and *Chaetoceros gracilis* showed mortality after 28 min of incubation at 80 mg/ml concentration (Peraman and Nachimuthu, 2019).

### Antioxidant Activity

Antioxidants are well-known bioactive chemicals that the human body needs to maintain health (Li et al., 2014; Yadav et al., 2016). They prevent further oxidation reactions from producing free radicals. Free radicals target essential macromolecules leading to cell damage and death and, as a result, cause a variety of serious chronic diseases such as cancer and atherosclerosis (Sailaja Rao et al., 2011; Mittal et al., 2014). A free radical is a molecular intermediate or byproduct of any biological pathway having an atomic unpaired electron. This unpaired electron results in high instability and reactivity of the free radical leading to oxidation or reduction of the biomolecules. Hydroxyl radicals, oxygen singlets, hydrogen peroxides, superoxide anion radicals, hypoclorites, nitric oxide radicals, peroxynitrite radicals, etc., are the frequently found free radicals. Life-leading molecules in the cells such as proteins, carbohydrates, lipids, and DNA or RNA molecules are being degraded by these free radicals (Young and Woodside, 2001; Mohammed et al., 2015). The major sources of the reactive oxygen species (ROS) are mitochondrial respiration, chloroplasts, xanthine oxidoreductase activity, dopamine breakdown, photosensitization reactions, etc. (Turrens, 2003; Berry and Hare, 2004; Moller et al., 2007; Miyazaki and Asanuma, 2008; Gill and Tuteja, 2010; Mohammed et al., 2015). Marine compounds have recently gained the attention of the pharmaceutical and food sectors as a source of potential antioxidants (Ngo et al., 2011). Seaweeds have substantial antioxidant properties that operate in chemical defense mechanisms to survive in harsh marine environments. The antioxidant capacity of brown seaweeds is higher than that of red or green seaweeds, according to many researches on several seaweed species (Jiménez‐Escrig et al., 2001; Matanjun et al., 2008; Cox et al., 2010). Many of the biological benefits of marine carotenoids such as anticancer, anti-obesity, and anti-inflammatory effects are linked to their capacity of scavenging ROS (Sachindra et al., 2007; Sachindra et al., 2010). FX is considered to be a useful antioxidant due to its unusual chemical structure, which includes an allenic bond, an epoxide group, and a hydroxyl group (Sangeetha et al., 2009). FX is a potent antioxidant that can quench hydroxyl radicals, superoxide radicals, and singlet oxygen and also prevent oxidative damage caused by H_2_O_2_ and ultraviolet B radiation (Heo and Jeon, 2009; Sachindra et al., 2010). Its pure form is susceptible to oxidation. Despite this, it was shown to be quite stable in the presence of co-existing antioxidants like polyphenol (Miyashita and Hosokawa, 2007).

The antioxidant effects of FX and its two metabolites (FXol and halocynthiaxanthin) were evaluated *in vitro* concerning singlet oxygen quenching abilities and radical scavenging. FX and FXol had stronger 2,2-diphenyl-1-picrylhydrazyl (DPPH) radical scavenging activity than halocynthiaxanthin. FXol had a higher 2,2′-azino-bis(3-ethylbenzothiazoline-6-sulfonic acid) (ABTS) radical scavenging activity than FX. Additionally, the antioxidant activity of FX against hydroxyl radical was greater than FXol and halocynthiaxanthin (Sachindra et al., 2007). Trans isomer exhibited stronger activity than cis-isomer. The antioxidant activity of FX against DPPH, hydrogen peroxide, superoxide anion, and reducing power reduced by 21.0, 16.0, 10.3, and 19.7%, respectively, when the percent of cis-isomer increased by 2% (Kawee-Ai et al., 2013). Under anoxic circumstances, FX equimolarly reacted with DPPH, while only one fraction of FX consumed DPPH under aerobic circumstances, and the degree of interaction varied with multiple attempts. Conversely, zeaxanthin, β-cryptoxanthin, β-carotene, lutein, and licopen scarcely reacted with DPPH under anoxic conditions (Nomura et al., 1997). The antioxidant activities of FX extracted from different algae against various active radicals are shown in Table 3.

**TABLE 3:** Antioxidant properties of FX extracted from various algae against free radicals.

Algae species containing FX	Active radicals	Activity	Ref
*Hijikia fusiformis*	DPPH	Scavenging activity	Yan et al. (1999)
*Undaria pinnatifida*			
*Sargassum* fulvellum			
*Cladosiphon okamuranus*	DPPH	Scavenging activity of FX and FXol extracted with methanol solvent	Mise et al. (2011)
*Cystoseria hakodatensis*	DPPH, peroxyl radical, ABTS, NO (nitric oxide)	Scavenging activity	Airanthi et al. (2011a)
*Sargassum* horneri	DPPH, peroxyl radical		
*Eisenia* bicyclis			
*Odontella aurita*	DPPH, ABTS	Scavenging activity of all-trans-FX isomer	Xia et al. (2013)
	DPPH, 12-DS and NB-LP	Reduction activity	Nishino, (1998)
*Undaria pinnatifida*	DPPH, ABTS, hydroxyl radical	Radical scavenging and singlet oxygen quenching abilities of FX and its two metabolites (FXol and halocynthiaxanthin)	Sachindra et al. (2007)
*Isochrysis galbana*	DPPH	Scavenging activity	Mousavi Nadushan and Hosseinzade, (2020)
*Sargassum duplicatum*	N.D	Inhibitory concentration measurement (IC_50_) and effect of different extraction methods (ethanol, methanol, and ethyl acetate solvent) on antioxidant activity	Savira et al. (2021)
*Phaeodactylum tricornutum*	DPPH, hydrogen peroxide, superoxide anion	Reduction activity, effect of different extraction methods (acetone, DMSO, ethanol, and 80% methanol solvent) on antioxidant activity, radical scavenging activity of FX with acetone extract, effect of trans- to cis-form ratio	Kawee-Ai et al. (2013)
*Phaeodactylum tricornutum*	DPPH	Effect of the different environments (anoxic and aerobic conditions) on scavenging activity, comparison of antioxidant activities with other carotenoids	Nomura et al. (1997)
*Sargassum siliquosum*	DPPH	Trolox equivalent antioxidant capacity, radical scavenging activity, ferric reducing antioxidant power (FRAP), effect of extraction parameters (temperature, time, and solvent to solid ratio)	Lim et al. (2018)
*Sargassum polycystum*			
*Sargassum fusiforme*	DPPH, hydroxyl radical	Effect of blanching treatments (hot water blanching, salt water blanching, microwave blanching, and steam blanching) on scavenging activity	Nie et al. (2021)

**FIGURE 1:**
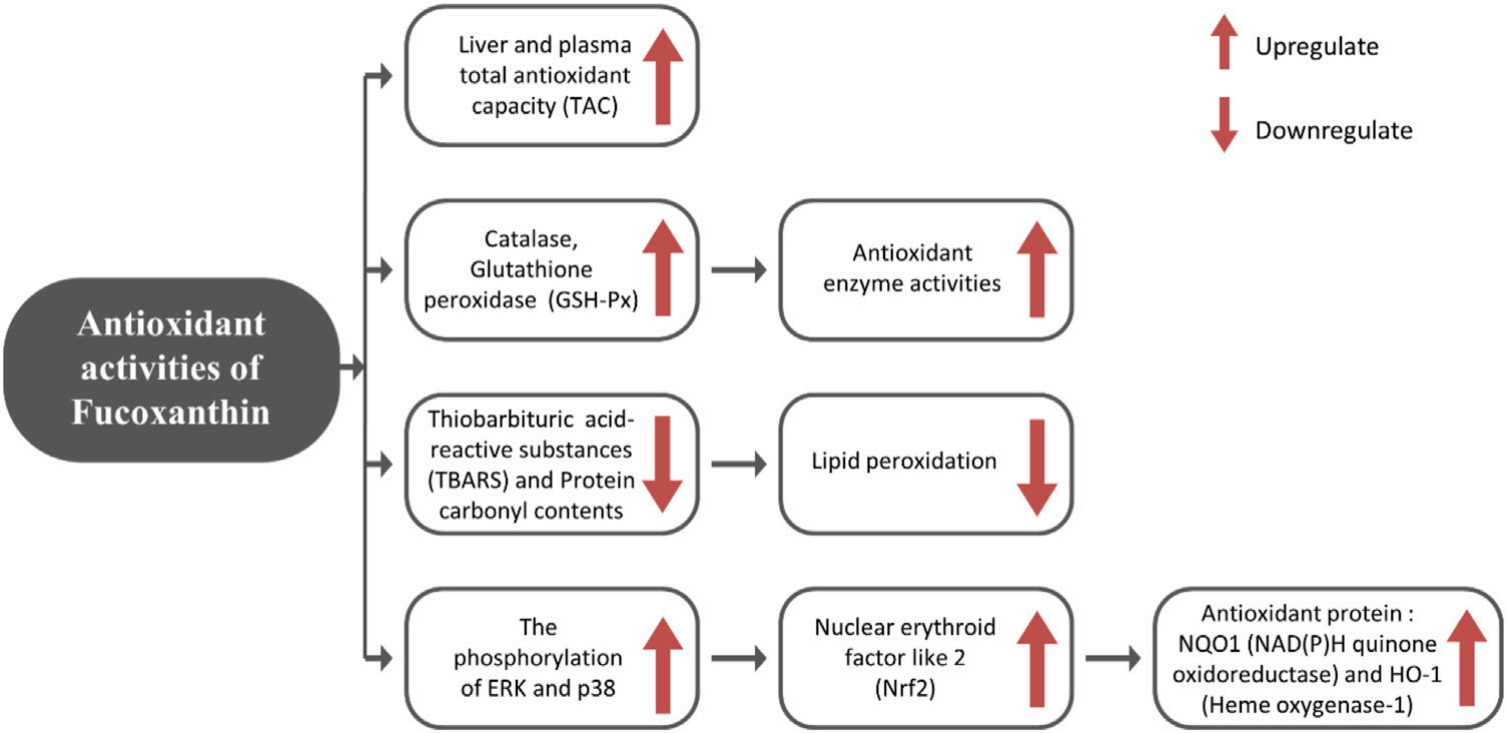
Role of dietary FX in antioxidant activity.

FX exerts its antioxidant impact through increasing antioxidant enzyme activities (catalase, gluthathione peroxidase (GSH-Px)) and total antioxidant capacity (TAC) of plasma, as well as enhancing mRNA expression of nuclear erythroid factor like 2 (Nrf2), which increases the synthesis of antioxidant proteins such as heme oxygenase-1 (HO-1) and NAD(P)H quinone oxidoreductase 1 (NQO1) (Liu et al., 2011; Ha et al., 2013). Nrf2 is a key controller of antioxidant protection responses that regulate the rate of essential detoxifying genes (Phase II genes) and antioxidants (Kwak et al., 2001). NQO1 and HO-1 are phase II genes that scavenge reactive nitrogen species (RNS), reactive oxygen species (ROS), xenobiotics, and detoxify electrophiles and also retain cellular reducing capacity (Cho et al., 2002). The antioxidant mechanism induced by FX is described in Figure 1.

### Anti-Inflammatory/Antiallergic Activity

Inflammation is a part of the complicated protective biological response of body tissues to fight against pathogens, damaged cells, and irritants (Chen et al., 2018). The inflammatory reaction may cause harm to healthy cells, tissues, and organs in the long run by generating superoxide anions, nitric oxide radicals. This may induce DNA damage, tissue death, and internal scarring over time (Jounai et al., 2012). The inflammatory responses are regulated by lipid mediators (prostaglandins and leukotrienes) and cytokines (Ross, 2002). Macrophages produce excessive pro-inflammatory cytokines like interleukin-1β (IL-1β), interleukin-6 (IL-6), tumor necrosis factor-α (TNF-α), and inflammatory mediators such as prostaglandin E2 (PGE_2_), nitric oxide (NO), and ROS, generated by activated cyclooxygenase (COX) and inducible nitric oxide synthase (iNOS) (Seon-Jin et al., 2003; Walsh et al., 2005; Yuan et al., 2006). Anti-inflammatory drugs work by decreasing these inflammatory elements and their mRNA expressions (Seon-Jin et al., 2003; Yuan et al., 2006). FX exhibited both anti-inflammatory and antiallergic potential in several *in vivo* and *in vitro* studies.

FX suppressed the inflammatory response of endotoxin-stimulated uveitis (EIU) (Shiratori et al., 2005) and dextran sulfate sodium (DSS)-stimulated colitis (Yang et al., 2020). So, FX can be used to control ulcerative colitis (UC), an inflammatory bowel disease. FX and Fxol can also suppress ear swelling, ear edema, and erythema (Khan et al., 2008a; Khan et al., 2008b; Sakai et al., 2011; Sugiura et al., 2016). This anti-inflammatory effect is comparable with epigallocatechin gallate (EGCG). FX inhibits mast cell degranulation by reducing the antigen-stimulated aggregation of high-affinity immunoglobulin E (IgE) receptors where mast cell degranulation signals activation, which were essential in inflammation and instant allergic responses (Sakai et al., 2009). Namkoong *et al.* investigated the anti-allergic mechanisms of FX fraction extracted from *Eisenia bicyclis* in an IgE–antigen complex (IgE/2,4-dinitrophenol (DNP)-BSA)-induced RBL-2H3 mast cells. In IgE/DNP-BSA-induced RBL-2H3 cells, they found that FX inhibited the release of β-hexosaminidase, transcriptional activation of nuclear factor kappa B (NF-κB), and phosphorylation of c-Jun N-terminal kinases (JNK) and extracellular regulated kinase (ERK). So, FX may aid in the prevention of allergic disorders including asthma and atopic dermatitis (Namkoong et al., 2012).

### Skin Protection

Skin serves as a primary protection against environmental elements, shielding the body from toxic chemicals, physical injury, pathogenic infiltration, and ultraviolet (UV) radiation. Ultraviolet B-ray (UVB) radiation exposure (280–315 nm) is the primary source of ROS and has been linked to a significant acute skin inflammatory response, which is defined by the activation of innate immune cells including macrophages and neutrophils to the dermis and epidermis (Duncan et al., 2009). Overexposure to UV also causes several cutaneous diseases such as laxity, pigmentation, erythema, wrinkling, and skin cancer. Recently, marine biological active compounds are becoming an important element of skincare products owing to their strong antioxidant and anti-inflammatory properties and their low toxicity (Berthon et al., 2017; Brunt and Burgess, 2018).

Keratinocytes are the most common kinds of cells (make about 90%) in the epidermis, the skin’s outermost layer. In human keratinocytes (HaCaT), FX enhances the messenger RNA (mRNA) expression and protein levels of glutathione synthetase (GSS), glutamate-cysteine ligase catalytic subunit (GCLC), phosphorylation of Akt (active form), and the nuclear translocation, phosphorylation of Nrf2. Additionally, FX promotes the binding of the antioxidant response element (ARE) sequence to Nrf2. Thus, FX restores glutathione (GSH) level that had been reduced by UVB irradiation (Zheng et al., 2014). FX shows a protective effect against UVB-induced skin photoaging by significantly suppressing UVB-induced matrix metalloproteinase-13 (MMP-13), vascular endothelial growth factor (VEGF) expression, and epidermal hypertrophy and thiobarbituric acid reactive substances (TBARS) in hairless mice (Urikura et al., 2011). FX lessened the mRNA expression of endothelin receptor A, prostaglandin E receptor 1 (EP1), p75 neurotrophin receptor (NTR), tyrosinase-related protein 1 (TYRP1), melanocortin 1 receptor (MC1R), COX-2, and tyrosinase activity. In this way, FX exhibits the inhibition of UVB-induced skin pigmentation and melanogenesis in melanoma (Shimoda et al., 2010). Filaggrin (Flg) promoter activity can be increased by FX. The protective properties of FX against UV-induced sunburn may be due to the production of filaggrin, which promotes the development of a skin barrier (Matsui et al., 2016).

### Anti-Obesity Activity

Obesity is a nutritional disorder, characterized by an excessive or abnormal accumulation of fat in the abdomen and viscera (Kopelman, 2000). It is the most common chronic disease, related to many environmental and genetic factors (Ichihara and Yamada, 2008; Durand et al., 2011; Albuquerque et al., 2017). Obesity increases the risk of many other serious diseases such as heart disease, type II diabetes, stroke, high blood pressure, fatty liver disease, gallbladder disease, high cholesterol, coronary atherosclerosis, sleep apnea, infertility, and certain cancers including breast, endometrial, and colon cancers (Matsuzawa et al., 2003; Fabbrini et al., 2010; Lee et al., 2013; Nigro et al., 2014). As a result, obesity control is a significant public health concern, and the development of non-toxic anti-obesity agents is crucial. According to many researches, FX can potentially inhibit obesity. Even a lower dose of FX can effectively decrease body weight (Tsukui et al., 2009; Woo et al., 2009; Jeon et al., 2010; Hu et al., 2012; Koo et al., 2019), body fat accumulation (Maeda et al., 2009; Maeda, 2015; Hitoe and Shimoda, 2017; Koo et al., 2019), and visceral fat-pad weight (Woo et al., 2009; Jeon et al., 2010; Muradian et al., 2015). Additionally, it lowers the size of adipocytes, the weight gain of white adipose tissue (WAT), while increases the weight of brown adipose tissue (BAT) (Maeda et al., 2005; Maeda et al., 2007a; Miyashita, 2009; Hosokawa et al., 2010; Hu et al., 2012; Miyashita, 2014; Maeda et al., 2015; Miyashita and Hosokawa, 2017) in high-fat (HF) diet-induced obese C57BL/6N mice, diabetic/obese KK-Ay mice, and C57BL/6J mice.

Several ways have been found that describe the anti-obesity effects of FX in experimental animals. Among them, *1*) regulation of lipid metabolism and *2*) effect on uncoupling proteins and adipocyte differentiation are thought to be the major anti-obesity mechanisms of FX.

### Regulation of Lipid Biosynthesis and Metabolism

Long-term imbalanced diets create a bad impact on lipid metabolism and cause visceral fat accumulation, resulting in obesity. FX affects lipid metabolism-related pathways and lowers the level of free fatty acids. It positively influences fatty acid β-oxidation, sirtuin 1 (Sirt1)/AMP-activated protein kinase (AMPK) pathway, lipolysis, and bile acid synthesis pathway and negatively influences lipogenesis and mevalonate pathway.

FX increases the fatty acid β-oxidation metabolism by enhancing the mRNA expression of β-oxidation-related acyl-CoA oxidase 1 (ACOX1) (Wu et al., 2015; Jia et al., 2016), carnitine palmitoyl-transferase 1(CPT1) (Woo et al., 2010; Chung et al., 2013; Jang and Choung, 2013; Gille et al., 2019), and peroxisome proliferator-activated receptor (PPAR)-alpha (Wu et al., 2014; Chang et al., 2018; Koo et al., 2019). Fatty acids are broken down to produce energy through the β-oxidation process. ACOX1 is a rate-regulating enzyme of the β-oxidation process in peroxisomes and also responsible for the breakdown of very long-chain fatty acids (Vluggens et al., 2010). Additionally, CPT1 plays a crucial role to control β-oxidation in mitochondria by converting acyl-CoA to long-chain acylcarnitine. It is suggested that increasing CPT1 could be used to develop novel obesity-prevention therapies (Schreurs et al., 2010). PPAR-alpha, a major controller of energy homeostasis, regulates the expression of genes involved in β-oxidation (Reddy and Rao, 2006; Cherkaoui-Malki et al., 2012; Ito et al., 2012; Ren et al., 2019). FX supplementation regulates fatty acid synthesis by significantly lowering the tmRNA expression of acetyl-CoA carboxylase (ACC) (Chung et al., 2013; Grasa-López et al., 2016; Chang et al., 2018) and fatty acid synthase (FAS) (Woo et al., 2009; Woo et al., 2010; Chung et al., 2013). ACC regulates the irreversible chemical reaction of acetyl-CoA to yield malonyl-CoA. This represents a building block for new fatty acid production and inhibits the fatty acid β-oxidation in the mitochondria (Hardie and Pan, 2002; Kim CW. et al., 2010). FAS is an essential enzyme that regulates the conversion of malonyl-CoA and acetyl-CoA to long-chain fatty acids (Park et al., 2019).

**FIGURE 2:**
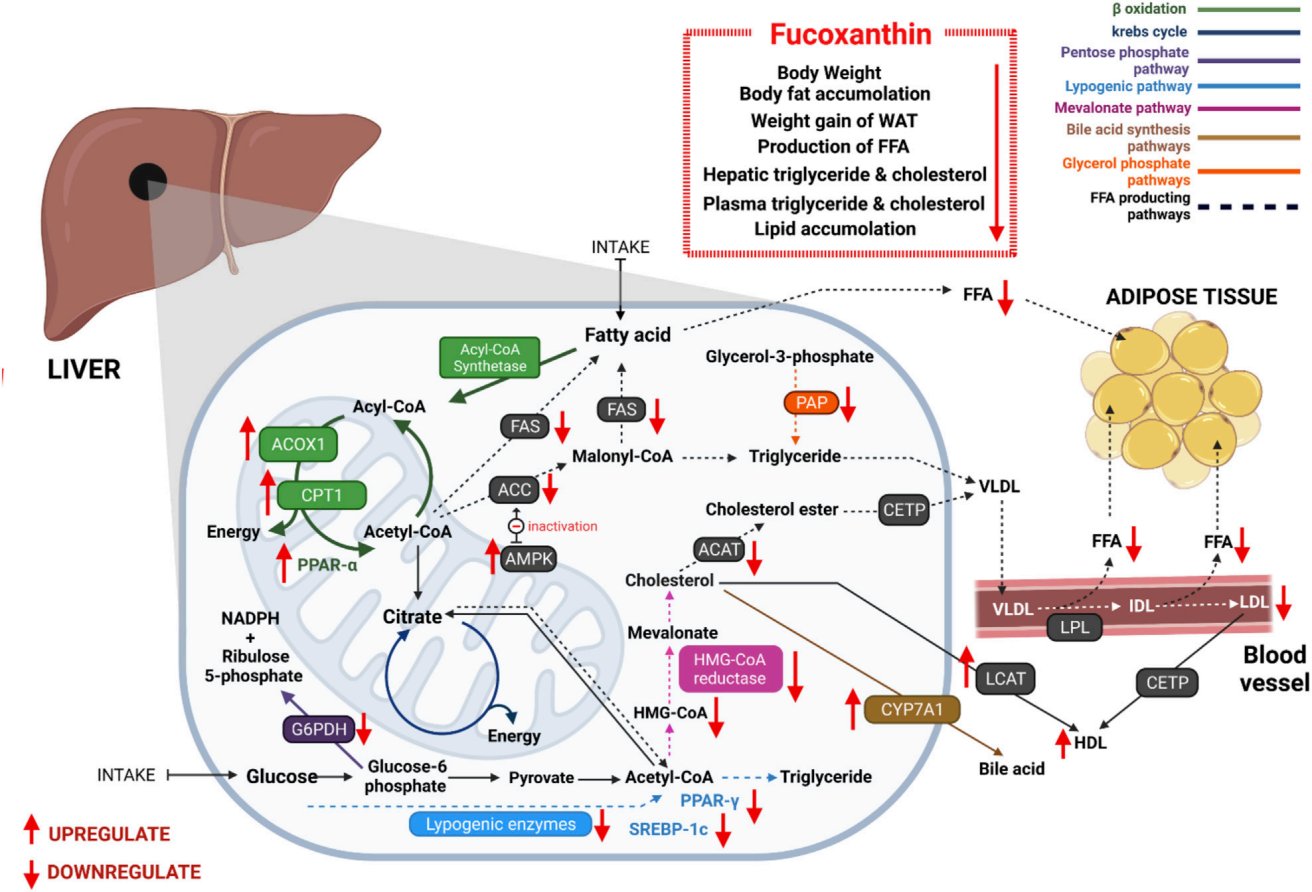
Antiobesity effects of FX through regulating lipid metabolism: FX significantly reduces free fatty acid synthesis *via* controlling many enzymes, which are associated with lipid metabolism. It promotes β-oxidation, bile acid synthesis pathway, and Sirt1/AMPK pathway and inhibits lipogenesis, mevalonate pathway, pentose phosphate pathway, and glycerol phosphate pathway. As a result, plasmatic and hepatic triglyceride and cholesterol concentrations are decreased. It reduces lipid accumulation in hepatic cells and adipose tissues.

All of these findings suggest that FX reduces triglyceride and cholesterol synthesis as well as the production of free fatty acids (FFAs). Thus, the level of hepatic and plasma triglycerides and cholesterol concentrations are decreased and fecal triglycerides and cholesterol concentrations are be increased by dietary FX (Jeon et al., 2010; Woo et al., 2010; Chung et al., 2013). Furthermore, both lymphatic triglyceride absorption and concentration in systemic circulation are reduced by FX and FXol, most likely owing to their inhibitory capacity on lipase activity in the gastrointestinal lumen (Matsumoto et al., 2010). Chang *et al.* (Chang et al., 2018) reported that FX impeded lipid peroxidation and inhibited lipid accumulation in FL83D hepatocytes. The effects of FX on lipid metabolism are shown in Figure 2.

### Effect on Uncoupling Proteins and Adipocyte Differentiation

According to recent studies, FX processes an anti-obesity activity mostly by stimulating the expression of uncoupling protein-1 (UCP-1) in WAT (Maeda et al., 2005; Miyashita, 2009; Gammone et al., 2014). UCP-1 is a critical molecule for metabolic heat production that is typically expressed exclusively in BAT. Adult individuals, on the other hand, have relatively minimal BAT and most of their fat is accumulated in WAT. Increased UCP-1 expression leads to increased energy expenditure, which helps to prevent excessive fat formation. Regulation of the mRNA expression of UCP-1 in tissues other than BAT by dietary constituents is considered an advanced discovery for effective obesity treatment. FX stimulates thermogenesis by increasing the level of energy produced as heat in fat tissue. When experimental mice were given *Undaria pinnatifida*, containing FX, UCP-1 protein and its mRNA expression were found in WAT. By boosting UCP-1 expression, 0.2% FX substantially reduced weight growth in mice (Maeda et al., 2005). FX and its metabolites promote UCP-1 in WAT, which causes fatty acid oxidation and heat generation (Kang et al., 2011). FX was also found to promote the β3-adrenergic receptor (Adrb3), which is responsible for thermogenesis and lipolysis. Through the lipolysis pathway, lipid triglycerides are hydrolyzed. This adaptive thermogenesis and lipolysis are seemed to play an important role in limiting weight gain and favoring weight reduction by increasing energy expenditure as heat.

### Antidiabetic Activity

FX has promising potential as a therapeutic drug for the treatment of type 2 diabetes and diabetes-related problems. In individuals with type 2 diabetes, glucose production is abnormally elevated. Adipose tissue plays an essential role in fat and glucose metabolism. Adipokines (cell-signaling proteins produced by adipose tissue) affect insulin sensitivity, glucose metabolism in liver, muscle, and adipose tissues. Unbalance WAT causes obesity as well as diabetes mellitus and cardiovascular diseases (Walker et al., 2007). Dietary FX inhibits the formation of WAT in obesity/diabetes mouse KK-Ay (Maeda et al., 2005).

Obesity raises the adipokine tumor necrosis factor-α (TNF-α), which causes the development of insulin resistance as well as diabetes (Nieto-Vazquez et al., 2008; Ye, 2013; Ren et al., 2019). Resistin, leptin, and adiponectin are also associated with insulin resistance (Kitagawa et al., 2004). Maeda *et al.* (Maeda et al., 2007b) reported that FX and fish oil mixture showed a remarkable antidiabetic effect in KK-Ay mice. Both 0.2 and 0.1% FX doses highly decreased plasma glucose and 0.2% FX reduced the plasma insulin level in KK-Ay mice. In addition, the mRNA expression of leptin in WAT and plasma leptin was decreased in the mice fed 0.2% FX and 0.1% FX with fish oil. Although the mRNA expression of tumor necrosis factor alpha (TNF-α) in WAT was remarkably lowered in the mice fed 0.2% FX diet, resistin and adiponectin were not affected by FX. Hepatic gluconeogenic enzyme activities are favorably linked with blood glucose level, while hepatic glucokinase activity is negatively associated (Tirone and Brunicardi, 2001). An excessively high rate of hepatic gluconeogenesis contributes to hyperglycemia in diabetes. FX supplements increased the hepatic glucokinase/glucose-6-phosphatase ratio and glycogen content in diet-induced obese mice (Park et al., 2011). Saturated fat consumption raises the hemoglobin A1c (HbA1c) level (Harding et al., 2001). The HbA1c level is a diabetes and glycemia complication risk indicator (Goldstein et al., 2004). Woo et al. (2010) observed that FX reduced the HbA1c level and blood glucose along with insulin and resistin concentrations in the plasma of HF diet-fed C57BL/6N mice. According to Jung et al. (2012)’s investigation, FX exhibited antidiabetic activity through inhibiting human recombinant aldose reductase (HRAR), rat lens aldose reductase (RLAR), protein tyrosine phosphate 1β (PTP 1β), and advanced glycation end-product (AGE) formation.

### Effect on Gut Microbes

Trillions of non-pathogenic symbiotic microbes have been found in the human. In humans, gut microbiota includes the most bacteria species when compared to other regions of the body (Quigley, 2013). Gut microbes aid in the breakdown of various indigestible nutritive compounds, such as cellulose, xylan, and digestion-resistant starch and increase nutrient diet digestion and absorption (Turnbaugh et al., 2006; Rowland et al., 2018). It suggests that nutritive compositions and gut microbes are interrelated and interact with each other. Changes in the group of gut microbes caused by age and other environmental variables (Diet) influence the condition of the host health (Maslowski and Mackay, 2011; Scott et al., 2013). Furthermore, intestinal microbes survive through interacting with their surroundings, which include the central endocrine system, nervous system, and immune system (Cheng et al., 2017; Colpitts et al., 2017; Velmurugan et al., 2017; Yissachar et al., 2017). High cholesterol, high blood sugar, weight gain, and other disorders can be caused by an imbalance of harmful and beneficial microbes in the intestines (Angelakis et al., 2012; Scott et al., 2015; Bo et al., 2017; Jastroch et al., 2020). For this reason, the alteration of gut microbiota by carotenoids has recently become a preferable research focus. Some recent studies show that FX modulates gut microbiota, which can be beneficial for human health.

A significant decrease in beneficial bacteria (such as *Bifidobacterium*, *Lactobacillus*, and butyrate-producing bacteria), as well as an increase in opportunistic pathogenic bacteria (such as Desulfovibrionaceae and Erysipelotrichaceae), can occur, which may cause further inflammation, bacterial and toxin translocations, and obesity (Turnbaugh et al., 2006; Zhang et al., 2010; Qin et al., 2012; Vrieze et al., 2012; Chang et al., 2015; Lecomte et al., 2015; Foster et al., 2016). Sun *et al.* (Sun et al., 2020) reported that FX can alleviate HF diet-induced gut microbiota dysbiosis. According to their 16S rRNA sequencing results, FX can significantly inhibit the growth of Firmicutes phyla (mainly Erysipelotrichaceae and Lachnospiraceae), which is linked with obesity and inflammatory responses (Turnbaugh et al., 2008; Delzenne and Cani, 2011; Remely et al., 2016; Zeng et al., 2016; Lippert et al., 2017; Sun et al., 2020; Zhou et al., 2020). Additionally, FX can increase the growth of *Bifidobacterium*, *Lactobacillus*/*Lactococcus*, and some butyrate-producing bacteria. Sun *et al.*’s (Sun et al., 2020) study also included that 0.05% FX dose with a normal diet enriched the abundance of *Enterococcaceae, Bacteroidetes*, *Bifidobacteriaceae, Ruminococcaceae*, *Bacteroidaes_S24-7_group*, and other strains. In particular, while alleviating HF diet-induced obesity, FX treatment increases *Anaerotruncus, Blautia obeum, Enterococcus durans, Streptococcus, Romboutsia, Lactobacillus equicursoris, Lactobacillus gasseri, Lactobacillus helveticus, Lactococcus lactis, and Lactococcus raffinolactis. Lactobacillus, Bifidobacterium,* and *Bacteroidaes_S24-7_group* inhibit obesity (Delzenne and Cani, 2011; Zhao L. et al., 2017; Shang et al., 2017). *Lactobacillus* can significantly decrease the WAT weight and body weight (Miyoshi et al., 2014; Shirouchi et al., 2016) and is capable of producing large quantities of lactic acid, which enhances gut microbial composition (Damodharan et al., 2016; Alok et al., 2017). Additionally, *Anaerotruncus*, *Blautia*, *Enterococcus durans*, and some genus in Ruminococcaceae family can possess anti-inflammatory activity, produce butyrate, and improve insulin sensitivity (Wang T. et al., 2012; Broecker et al., 2016; Cook et al., 2016; Kanda et al., 2016; Liu et al., 2018).

Various studies ensure that FX can be used to improve health in many ways by controlling the growth of gut microbes. But the mechanisms of interaction between FX and gut microbes are unknown, and further studies are needed to find out the gut microbe modulation process of FX.

### Hepatoprotective Activity

Various studies have determined and showed the optimistic effects of FX on liver protection in rodents including mice, rats as well as in human body environment. Enhancing the oxidation of fatty acids and decreasing the substrates for triacylglycerol synthesis can help in fatty liver diseases (Park et al., 2011). FX has been observed to notably decrease hepatic lipid contents as well as to increase feces weight and fecal lipids due to the inhibition of lipid absorption in C57BL/6N mice administered with HF diet (Woo et al., 2010). The enzymatic reactions associated with hepatic fatty acid synthesis were minimized by FX in liver, reducing the accumulation of hepatic lipid content in HF-fed mice, as described by Park *et al.* (Woo et al., 2010; Park et al., 2011).

Docosahexaenoic acid (DHA), an essential ω-3 functional polyunsaturated fatty acid, stimulates the β-oxidation of hepatic fatty acid in liver as well as minimizes hepatic enzyme activity employed in fatty acid synthesis (Maeda et al., 2008; Woo et al., 2010; Park et al., 2011). FX supplementation effectively reduced the hepatic lipid concentration and plasma triglyceride concentration in C57BL/6N mice (Woo et al., 2010). The activities of G6PD, ME, FAS, and PAP hepatic lysogenic enzymes were inhibited by FX, as found in this study. The contribution of FX and FXol in elevating DHA in KK-*A* ^*y*^ mice liver was first described by Tsukui *et al.* (Tsukui et al., 2007). The same contribution of FX was also reported in C57BL/6J mice (Tsukui et al., 2009). In addition, Airanthi *et al.* (Airanthi et al., 2011b) reported the elevation of DHA and arachidonic acid (ARA) in KK-*Ay* mice when served with brown seaweed lipids. In the first study, the small intestinal level of the acid was unchanged. As the enhanced level of ARA (ω-6) has been noticed in FX-fed mice, FX is thought to alter the metabolic pathway of ω-3 and ω-6 fatty acids.

Zheng *et al.* (Zheng et al., 2019) showed the effective role of FX in alcohol-induced liver damage *in vivo*. Its protective effect on the gastric mucosa was also evaluated in this study. Activities of the two enzymes, serum aspartate transaminase (AST) and alanine transaminase (ALT), were lowered by FX treatment, indicating its positive effect on alcohol-induced liver injury (Mohibbullah et al., 2018; Zheng et al., 2019). Fat deposition in alcohol-induced liver was effectively reduced by FX. Immoderate oxidation of hepatocytes due to alcohol intake might also be prevented by FX treatment, improving the antioxidant capacity of the hepatic cells. FX also restricted the pro-inflammatory factor secretion, resulting in lower inflammatory response in alcohol-induced liver injury.

### Neuroprotective Activity

Neuronal dysfunction or demission due to central nervous system (CNS) injury induces neuronal apoptosis and degradation. Bioactive natural and synthetic compounds that induce specific mechanisms to protect neuronal cells from incidents occurred by CNS injury are known to have neuroprotective effects (Tucci and Bagetta, 2008; Zarros, 2009). Having several side effects including drowsiness, anxiety, tiredness, dry mouth, etc., synthetic compounds are often postponed by researchers (Narang et al., 2008; Pangestuti and Kim, 2010)**.** Several studies have been conducted to find out the contributions of FX in neuroprotection and revealed significant mechanisms that can help in developing essential pharmaceuticals.

Several studies have revealed the effects of FX in lipopolysaccharide (LPS)-induced RAW 264.7 cells and amyloid beta (Aβ)-induced BV-2 microglia on suppressing the production of NO, ROS, and pro-inflammatory cytokines (Shiratori et al., 2005; Kim N. W. et al., 2010b; Heo et al., 2012; Pangestuti et al., 2013; Choi et al., 2016)**.** A study conducted by Dong Zhao et al. (2017) has proved the dose-dependent activity of FX in LPS-activated BV-2 microglial cells. FX inhibited the protein and mRNA expression of pro-inflammatory mediators involving NF-κB and mitogen-activated protein kinase (MAPK) pathways involving JNK, ERK, and p38 in LPS-activated microglia resembling the finding of previous studies (Shiratori et al., 2005; Kim N. W. et al., 2010b; Pangestuti et al., 2013; Choi et al., 2016). They (Zhao D. et al., 2017) came to a conclusion that FX can be a promising agent arresting neuro-inflammatory diseases and protecting neuronal cells during several CNS injuries.

Ischemic/reperfusion (I/R) injury is considered as the most susceptible disease of brain cells (Lee et al., 2000). FX notably elevated B-cell lymphoma 2 (Bcl-2) expression and alleviated the expression of Bcl-2-associated X protein (Bax). It inhibited caspase-3 in brain tissue and OGD/R-treated neurons (Hu et al., 2018). All these findings suggest a potential role of FX as an anti-apoptotic agent in cerebral I/R injury. Activated Nrf2 possibly protects brain from I/R injury, whereas FX might induce Nrf2 and HO-1 activation, attenuating oxidative stress that is absent from cerebral I/R injury (Zhang et al., 2017; Hu et al., 2018). The overall findings on the FX contribution on neuronal cells define it as a significant neuroprotective agent and a great promise in future therapeutics of neurodegenerative diseases.

### Antiangiogenic Activity

Formation of new blood vessels involving migration, growth, and differentiation of endothelial cells is known as the angiogenesis process (Carmeliet, 2003). Angiogenesis is a highly regulated process that occurs in standard physiological activities including embryogenesis, ovary cycling, and wound healing. Rheumatoid arthritis, tumor metastasis, diabetic retinopathy, and several inflammatory diseases are assisted by uncontrolled angiogenesis (Kirk et al., 2004). Prevention of angiogenic abnormality can help defending several diseases as well as pathological emergency conditions. The effects of FX on resisting unwanted angiogenesis have been evaluated by Sugawara *et al.* (Sugawara et al., 2006) in cultured human umbilical vein endothelial cells (HUVECs) and in the rat aortic ring. When a dose of 10 μM FX is applied, significant suppression HUVEC proliferation has been noticed in this study, but FX showed no effect on HUVEC chemotaxis. Differentiation of endothelial progenitor cells into endothelial cells was also significantly suppressed by FX, and the formation of new blood vessel was also restricted. Moreover, FX limited the tube length of endothelial cells. In the experiment on rat aortic ring, the outgrowth of microvessel was effectively suppressed by FX and FXol in *in vivo* and *ex vivo* models of the rat aortic ring. All these findings suggest possible roles of FX in inhibiting or reducing diseases involving the unregulated angiogenesis process.

### Anticancer Effects

In 2020, approximately 10 million people died of none other than the second leading cause of death in mankind, cancer (World Health Organization, 2021). It has been known as a deadly disease derived from the uncontrolled cell division of organ tissues and spreads through invasion and subversion of normal tissues (Evan and Vousden, 2001). In the mob of various cancer types, lung cancer, breast cancer, prostate cancer, and colorectal cancer are the most threatening to human in this era (Bray et al., 2018). Poor diagnosis of cancer and low outcomes from conventional chemotherapy indicate the need of new approaches to reduce cancer-induced mortality rate and prevent neoplastic diseases (Sporn and Suh, 2000). Many natural compounds, specially antioxidative compounds such as carotenoids, exhibit anticancer activity and prevent carcinogenesis (Rokkaku et al., 2013; Bolhassani, 2015; Soares Nda et al., 2015; Ávila-Román et al., 2021). FX is now considered to be an effective compound in cancer therapies depending on tumor cell types and stages (Torres et al., 2020). Apoptosis is considered to be one of the six fundamental hallmarks of cancer and is a crucial factor to target for anticancer therapeutics and drugs (Brown and Attardi, 2005). FX is investigated in several cancer models, demonstrating apoptosis and cell-cycle arrest induced by the compound in cancer inhibition (Table 4) (Mills et al., 2018). The ability of FX to scavenge free radicals makes it a crucial modulator of carcinogenesis.

**TABLE 4:** Inhibitory effect of FX on different cancer types and its underlying mechanism.

Cancer type	Target cell lines	Inhibitory activity	Mechanism	Ref
Lung cancer	A549, H1299, MRC-5	Induces cell cycle arrest, nuclei fragmentation	Upregulates p21^waf1/cip1^, p53, Fas, PUMA, capase-3 or caspase-8 and downregulates Bcl-2	Moreau et al. (2006), Mei et al. (2017)
Liver cancer	HepG2, SK-Hep-1	Induces G0/G1 cell cycle arrest	Downregulates cyclin D	Das et al. (2008), Liu et al. (2009)
Gastric cancer	MGC-803, SGC-7901, BGC-823	Induces cell cycle arrest at S phase and G2/M phase	Upregulates caspase-3 and downregulates Bcl-2, STAT3, cyclinB1	Yu et al. (2011), Yu et al. (2018), Zhu et al. (2018), Xiao et al. (2020)
Breast cancer	MCF-7, MDA-MB-231	Induces apoptosis	Downregulates VEGF-C, VEGFr-3, NF-κB, Akt, P13K, translocates Ca^2+^ from ER to cytoplasm	Hou et al. (2014), Wang et al. (2019)
Leukemia	HL-60, K562, TK6	Inhibits cancer cell proliferation, induces apoptosis	Upregulates caspase-3/7/9, generates ROS, and downregulates Bcl-xL, cleaves PARP	Kotake-Nara et al. (2005c), Kim et al. (2010b), Hosokawa et al. (1999)
Lymphoma	BCBL-1, TY-1	Induces G1 cell cycle arrest and apoptosis	Downregulates NF-κB, AP-1, P13K	Yamamoto et al. (2011)
Glioma	U87, U521, B16-F10	Induces apoptosis	Upregulates caspase-3/9, Bax and downregulates MMP-2, MMP-9, Bcl-2	Nabeshima et al. (2002), Liu et al. (2016b), Chung et al. (2013)
Neuroblastoma	GOTO	Induces G0/G1 cell cycle arrest	Downregulates N-myc	Asai et al. (2004)
Colon cancer	Caco-2, DLD-1, HT-29, WiDr, HCT116	Induces DNA fragmentation, apoptosis, G0/G1 cell cycle arrest	Upregulates p21 and downregulates Bcl -2	Hosokawa et al. (2004), Das et al. (2005)
Cervical cancer	HeLa, SiHa, CaSki	Induces apoptosis	Upregulates Bax and downregulates P13K, Akt, NF-κB, Bcl-2	Jin et al. (2018)
Urinary bladder cancer	EJ-1, T24	Inhibits cell proliferation, induces apoptosis	Upregulates caspase-3, p21 and downregulates CDK-2, CDK-4, cyclin D1, cyclin E	Zhang et al. (2008), Wang et al. (2014)
Prostate cancer	PC-3, DU145, LNCaP	Induces apoptosis, G1 cell cycle arrest	Upregulates caspase-3, Bax and downregulates Bcl-2	Kotake-Nara et al. (2001), Kotake-Nara et al. (2005a), Yoshiko and Hoyoku, (2007)

### Lung Cancer

Inhibition of nasopharyngeal carcinoma cell proliferation by FX is described by Long *et al.* (Long et al., 2020). Non-small-cell lung cancer (NSCLC) is also prevented by FX at a certain dose (Mei et al., 2017). The cell lines of NSCLC, A549, and H1299 were inhibited by this compound. It upregulated p21^waf1/cip1^, p53, Fas, p53 upregulated modulator of apoptosis (PUMA), caspase-3 or caspase-8, and downregulated Bcl-2 in favor to cell-cycle arrest of the tumor cells. Moreau *et al.* (Moreau et al., 2006) showed that FX can trigger apoptosis to A549 and NSCLC-N6 cell lines mediating nuclei fragmentation and formation of apoptotic bodies. Human fetal lung fibroblast MRC-5 was also inhibited by FX (Kotake-Nara et al., 2005b). In a recent study, FX exerted the sensitivity of lung cancer cell lines to Gefitinib (Ming et al., 2021). FX inhibited several pathways and the expression of several proteins including fibronectin, N-cadherin, Snail, Twist, MMP-2, PI3K, p-AKT, and κκB, and increased the TIMP-2 expression resulting in the inhibition of cancer cell migration and invasion in *in vitro* analysis.

### Liver Cancer

Liver cancer is considered one of the threatening cancers all over the world. The malignant tumor of liver cancer can be categorized under primary and secondary states. The active role of FX in inhibiting diethylnitrosamine (DEN)-induced liver cancer has been found in rat models (Jin et al., 2019). DEN increases lipid oxidation; hence, inhibiting this compound may reduce carcinogenic processes (Schneider et al., 2012; Ramalingam and Vaiyapuri, 2013; Ding et al., 2017)**.** FX effectively restored normal body weight, liver enzyme concentration, antioxidant enzyme concentration, stress markers, serum albumin, and serum bilirubin in this study (Jin et al., 2019). Das *et al.* (Das et al., 2008) showed that FX-rich fraction (FxRF) from crude methanolic extracts (CMEs) of *C. calcitrans* induced cytotoxicity to liver cancer cell line, HepG2. FX induced the G0/G1 cell-cycle arrest and inhibited the expression of cyclin D; hence, the viability of HepG2 cells was significantly diminished by 25 μM of this carotenoid. The same outcomes were studied by Foo et al. (2019), where cytotoxicity was induced by CME and FxRF to HepG2 cells. Human hepatoma SK-Hep-1 cell proliferation was also inhibited by FX but the compound supported the growth of BNL CL.2 cell, the murine embryonic hepatic cell (Liu et al., 2009).

### Gastric Cancer

A study documented the reduction activity of FX on mRNA, myeloid cell leukemia (Mcl-1) expression, and signal transducer and activator of transcription 3 (STAT3) proteins in SGC-7901 and BGC-823 cell lines of human gastric cancer (GC)-inducing cell-cycle arrest in the S phase and mediating apoptosis at the G2/M phase (Yu et al., 2011). In the same study, FX was observed to induce cell-cycle arrest of MGC-803, the human gastric adenocarcinoma cells in the G2/M phase, and also induced apoptosis of the cells. FX reduced CyclinB1 and surviving expression in MGC-803 cells. A further analysis on the reduction of cell viability and proliferation of human gastric adenocarcinoma BGC-823 and SGC-7901 showed the minimizing effect of FX on the corresponding cells (Yu et al., 2018). Mcl-1 is a protein from Bcl-2 family, which induces anti-apoptotic activity. Being a target protein of the TNF-related apoptosis-inducing ligand (TRAIL) signaling pathway, Mcl-1 is a significant target for anticancer drugs (Xiao et al., 2020). STAT3 induces chronic inflammation and acts as a helping factor in tumor generation. Inhibition of these proteins indicated the anticancer role of FX on GC cell lines. FX can be used in gastric adenocarcinoma therapy as it acts safely to the healthy cells. FX downregulated several key proteins including STAT3 and cyclin B1 in MGC-803 cells. FX also induced the G2/M phase apoptosis and cell-cycle arrest in the same study (Yu et al., 2011). In another study, FX upregulated beclin-1 and LC3 expressions, cleaved caspase-3, downregulated Bcl-2 expression, and consequently reduced the cell viability of SGC-7901 cells (Zhu et al., 2018). All these findings prove FX as a significant anti-GC agent.

### Breast Cancer

Breast cancer is the most threatening disease to women, which arise from the malignant tumor of the epithelial tissue of the breast. Inhibition of the phenotype of MDA-MB-231 cells, the malignant human breast cancer cell by FX has been demonstrated in a study (Wang et al., 2019). FX significantly reduced tumor-induced lymphangiogenesis in this study. NF-κB, vascular endothelial growth factor (VEGF)-C, VEGFr-3, phosphorylated P13K, and phosphorylated Akt levels are efficiently reduced by FX. *In vivo* and *in vitro* analyses with the MDA-MB-231 breast cancer model showed the reduction effect of FX on microlymphatic vessel density (micro-LVD) in mice and altogether suggests FX to be a potential component of anti-lymphagiogenic drugs applied in anti-malignant treatments of breast cancer patients. Hou et al. (2014) showed the damaging effect of FX on the endoplasmic reticulum (ER) membrane. Translocation of Ca^2+^ from ER to cytoplasm is induced by FX in MCF-7 cells, resulting in apoptosis of the cell. The expression of the SOX9 transcriptional protein is minimized by FX in MDA-MB-231 cells. In combination with adriamycin, oxidative stress-mediated apoptosis was initiated by this compound in breast cancer cells (Vijay et al., 2018).

### Leukemia and Lymphoma

Almeida et al. (2018) documented the role of FX in chronic myeloid leukemia (CML), which is the most recurring type of persistent leukemia and is developed by active proliferation of bone marrow hematopoietic stem cells. The authors investigated the FX activity through the *in vitro* analysis of K562 and TK6, the two human leukemia cell lines, at an isolated and a combined form with imatinib (Imat) and doxorubicin (Dox), which are known as anticancer drugs. FX notably inhibited the clonal proliferation of K562 and TK6 and also worked well in combination with imatinib and doxorubicin (Almeida et al., 2018). Investigating the underlying apoptotic mechanism of FX, Kil-Nam Kim et al. (2010a) found that ROS generation, Bcl-xL inactivation, caspase-3 and caspase-7 activation, and poly (ADP-ribose) polymerase (PARP) cleavage were induced by FX, and as a result, apoptotic action toward HL-60 was mediated by this compound. Ishikawa et al. (2008) examined the effect of FX and FXol on adult T-cell leukemia (ATL). ATL is a deadly malignant cancer of T lymphocytes. It develops from the infection of human T-cell leukemia virus type 1 (HTLV-1) and is incurable (Nicot, 2005). In comparison with β-carotene and astaxanthin, FX showed a stronger anti-ALT effect, inducing apoptosis to cancer cells (Ishikawa et al., 2008). Hosokawa et al. (1999) also documented the apoptosis induction of FX on human leukemia cells (HL-60). FX involved caspase-3, caspase-8, and caspase-9 activation to induce apoptotic pathways in cancer cells (Hosokawa et al., 1999).

Among the non-Hodgkin’s lymphoma, primary effusion lymphoma (PEL) is a serious cancer derived from the infection of human herpesvirus 8. PEL cell lines including TY-1 and BCBL-1 are suppressed by FX inducing G1 cell-cycle arrest and caspase-dependent apoptosis. FX suppressed the PEL cell growth in xenografted mice, indicating its anti-PEL therapeutic effect (Yamamoto et al., 2011). These findings indicate that FX can act significantly against leukemia and lymphoma.

### Roles in Glioma

Liu et al. (2016b) documented the cytotoxicity of FX to U87 and U521 cell lines, which are known as grade IV glioma or glioblastoma multiforme (GBM) (Rao, 2003; Dunbar and Yachnis, 2010). Despite being the most common primary tumor of CNS, the treatment of glioma including radiotherapy or chemotherapy often fails to reach a favorable outcome. FX inhibited the migration of U87 and U251 cells and their evasion through matrigel membrane as found in *in vitro* analysis by using the scratch wound healing assay and the trans-well assay, respectively (Liu et al., 2016b). For levels of caspase-3, caspase-9, etc., apoptotic proteins are increased by FX. In U87 and U251 cells, the levels of matric metalloproteinases 2 (MMP-2) and MMP-9 were decreased by FX, which proves the crucial role of FX in inhibiting evasion and metastasis of tumor cells (Nabeshima et al., 2002). Moreover, migration and evasion of murine B16-F10 melanoma cells were attenuated by FX, resulting in reduced actin fiber formation and reduced expression of MMP-9 level (Chung et al., 2013). A phosphoinositide 3-kinase (PI3K), Akt, and mammalian target of rapamycin (mTOR) protein inhibit cell apoptosis favoring tumor growth. FX effectively inhibits these anti-apoptotic proteins, increases Bcl-2 associated X (Bax) expression, and decreases Bcl-2 expression and therefore acts as an anti-glioma agent and can be utilized in cancer therapeutics (Liu et al., 2016b).

### Colon Cancer

Among the gastrointestinal cancers, colon cancer is the second most frequent. Hosokawa et al. (2004) described the inhibitory activity of FX against the proliferation of human colon cancer cell lines including Caco-2, DLD-1, and HT-29 cells mediating the fragmentation of DNA. This compound remarkably induced apoptosis to the colon cancer cell lines and reduced cell viability at a dose- and time-dependent manner. Interestingly, β-carotene and astaxanthin did not show any adverse effect on Caco-2 cells. FX decreased the level of Bcl-2 proteins, which are anti-apoptotic factors. In another study, several cancer-inducing incidents including colon damage were diminished on the FX treatment (Terasaki et al., 2020). Tumor growth was inhibited, and anoikis pathway was promoted in the carcinogenic mouse model by FX treatment. Moreover, mouse colon carcinogenesis was inhibited by FX that was induced by 1,2-dimethylhydrazine in an *in vivo* study (Kim et al., 1998). Das et al. (2005) observed the anti-proliferative effect of FX on HCT116 and WiDr cell lines, inducing the G0/G1 cell-cycle arrest by upregulating the p21^WAF1/Cip1^, the cyclin-dependent kinase (CDK) inhibitory protein, and retinoblastoma protein (pRb). The initiation crypt foci, known as a preneoplastic colon cancer marker was strongly inhibited in the mice model by FX. In a study, polyp formation was significantly inhibited by FX administration. Moreover, anoikis-like cells of colonic mucosal tissue was upregulated by this compound in mice models treated with azoxymethane and DSS and prevented colorectal tumors (Terasaki et al., 2019). Kotake-Nara et al. (2005b) showed that FX reduced the cell viability of Caco-2 and HCT116 cell lines. Terasaki et al. (2021) investigated the efficacy of FX on azoxymethane or DSS-induced colorectal cancer and found its inhibitory effect in mice on the multiplicity of colorectal adenocarcinoma. FX reduced *Bacteroidlales* and *Rikenellaceae* and increased *Lachnospiraceae,* which indicated a relation between the alteration of gut microbiota and suppression of colorectal cancer in the experimental mice.

### Cervical Cancer

The increasing rate of cervical cancer either induced by human papillomavirus (HPV) infection or other factors and its upgoing incidence is threatening the women’s physical and mental health. HPV infection is the most common sexually transmitted infection (STI), which transmits through skin-to-skin contact and causes cervical cancer. FX works in combination with the TRAIL pathway and induces apoptosis of HeLa, SiHa, and CaSki, the cervical cancer cell lines by targeting the signaling pathway including P13K, Akt, and NF-κB. FX enhanced Bax expression as well as decreased the Bcl-2 expression in SiHa cell, which induce apoptosis as an outcome (Jin et al., 2018).

### Roles in Other Cancers

Bladder cancer is a serious and the most expensive cancer to treat. FX reduces human urinary bladder cancer cell EJ-1 viability and induces apoptosis (Zhang et al., 2008). It inhibits EJ-1 proliferation and causes the increased percentage of hypodiploid cells and DNA ladder and activates caspase-3 activity. Human bladder cancer cell line T24 is also inhibited by FX, as described in a study (Wang et al., 2014). A CDK-inhibitory protein, p21, was increased and CDK-2, CDK-4, cyclin D1, and cyclin E were decreased by FX, as described in this study. Kotake-Nara et al. (2001) and Kotake-Nara et al. (2005a) showed the apoptotic effect of FX on PC-3, DU145, and LNCaP, the prostate cancer cell lines. FX inhibited PC-3 cell line with a 50% inhibitory concentration of 3 μM (Asai et al., 2004). The viability of PC-3, DU145, and LNCaP was minimized to 14.9, 5.0, and 9.8%, respectively, on the FX treatment (Kotake-Nara et al., 2001). FX interestingly reduced the level of Bax and Bcl-2 proteins in PC-3 cells inducing apoptosis in a different way from mechanisms mentioned in other studies (Kotake-Nara et al., 2001; Kotake-Nara et al., 2005a). FX inhibited cell growth of DU145 by inducing the G1 cell-cycle arrest (Yoshiko and Hoyoku, 2007).

Duodenal and skin carcinogenesis in mice was effectively blocked by FX, inducing anti-oxidant activity, cell-cycle arrest, and apoptosis (Liu et al., 2009; Liu et al., 2011; Wang J. et al., 2012). Mouse melanoma cell line B16 was also inhibited by FX (Kotake-Nara et al., 2005b). It prevented duodenal carcinogenesis in N-ethyl-N′-nitro-N-nitrosoguanidine-induced mice (Okuzumi et al., 1993). Moreover, the growth of human neuroblastoma cell line GOTO was inhibited, cell cycle was blocked at the G0/G1 phase, and N-myc gene expression was reduced by FX (Okuzumi et al., 1990). In several cases, FXol, the metabolite of FX (Asai et al., 2004) showed higher anticancer activity and better efficiency in cancer cells including Caco-2 and MCF-7 cells. Dietary FX converts to FXol and works in anticancer activities.

Despite a huge number of studies that prove the anticancer/anti-tumor activity of FX, its core mechanisms to induce tumor death are not clearly understood yet (Yoshiko and Hoyoku, 2007). Resting the apoptosis and cell-cycle arrest by FX, this compound may have several other mechanisms, which should figure out in future studies through investigations in different animal models against different types of malignancies.

### Bone-Protective Activity

**FIGURE 3:**
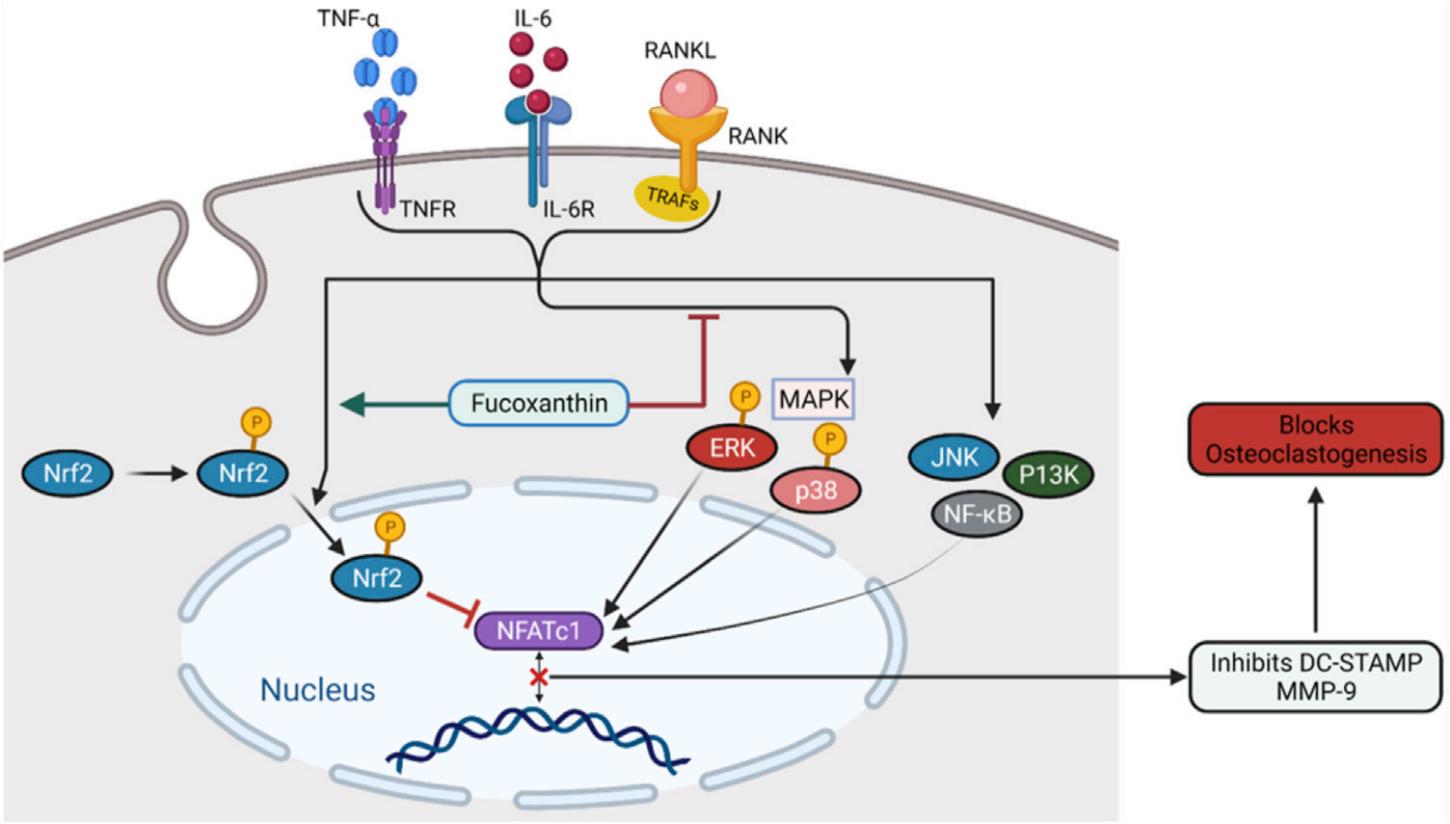
The mechanism of FX inhibiting osteoclastogenesis. FX inhibits ERK and p38, promotes the nuclear translocation of Nrf2, and downregulates NFATc1.

Bone is much susceptible to aging as age-related losses include severe problems such as bone decomposition, bone dysfunction, deterioration of bone structure, etc. (Raisz and Rodan, 2003). Postmenopausal women lack ovarian hormone E2 and elderly women deal with possibilities of osteoporosis (Khosla and Riggs, 2005; Zhang et al., 2011; Jilka, 2013; Khosla, 2013; Tabatabaei-Malazy et al., 2017). Drugs used to treat osteoporosis including bisphosphonate, estrogen, and anti-RANKL antibodies exert many side effects on human body (Wysowski, 2009; Rhee et al., 2012; Khan et al., 2015). Guo L. et al. (2020) studied the roles of FX in ovariectomy-stimulated osteoporosis in female Sprague Dawley (SD) rats. Administration of oral doses of 20 and 40 mg/kg FX for 16 weeks reversed the increase of weight and urine mass due to ovariectomy. Moreover, FX improved the bone mineral content and bone density and therefore provided protection against osteoporosis. In a study investigating medicinal effects of FX on alveolar bone resorption in the ligature-induced periodontitis mouse model, significant reduction of RANKL-positive osteoclasts has been reported (Kose et al., 2016). This result suggests the bone-protective effect of FX against osteoclast-related diseases. Ha et al. (2021) conducted the *in vitro* analysis on soluble receptor activator of NF-κB ligand or tumor necrosis factor-α (TNF- α)/interleukin-6 (IL-6)-stimulated RAW264.7 cell. FX significantly suppressed RAW264.7 differentiation and inhibited bone resorption. It suppressed ERK activation, p38 kinase activation, and initiated nuclear translocation of phospo-Nrf2, as illustrated in Figure 3. These findings prove the FX to be a possible therapeutic in the treatment of osteoclastogenesis having no immerse effect on bone formation.

### Eye-Protective Activity

Uveitis is a known inflammation of eye, specifically the inflammation of the middle layer of the eye wall tissue, called uvea. Several inflammatory signs including eye redness, foggy vision and pain rise up suddenly, and worsening of the situation can result in severe problems including vision loss (Chang and Wakefield, 2002; Wakefield and Chang, 2005; Bose et al., 2016). Development of lipopolysaccharide-induced uveitis was efficiently suppressed by FX in male Lewis rats, as described in a study by Shiratori et al. (2005). This study reveals the anti-ocular inflammatory activity of FX and can be utilized in future therapeutics. Among the excellent properties of FX, one is that it can absorb spectrum ranging from 350 to 550 nm having 450 nm of maximum absorption peak (Yan et al., 1999; Xiangyong et al., 2014). Between the range of visible light, blue light with a wavelength of 400–500 nm actively damages retinal tissues due to its strong energy and high penetrating power into the cells and organelles (Wenzel et al., 2005; Roehlecke et al., 2009). Liu et al. (2016a) described the function of FX in preventing light-induced retinal damage through *in vitro* and *in vivo* analyses. FX efficiently protected retinal pigment epithelium (RPE) cells from the damaging effect of visible light as per *in vitro* analysis. Moreover, the *in vivo* analysis described a significant role of FX in preventing retina from photoinduced damage. In these cases, FX showed better result than anthocyanins, lutein, and zeaxanthin, which are known as good ingredients for eye health care (Liu et al., 2016a).

## Conclusion

Fucoxanthin, a marine carotenoid with multiple disease-preventive qualities, is a crucial element on which a large number of researches are going on. Although the physiological benefits of carotenoids have received less attention, FX has recently gained a lot of interest owing to its significant anticancer and anti-obesity properties. Additionally, it prevents obesity-related diseases such as type 2 diabetics, cardiovascular disease, hepatic diseases, etc. FX exhibits anti-inflammatory and antioxidative effects, and through these activities, it inhibits allergic reactions and UVB irritations. It also shows remarkable organ-protective effects including bone protection, liver protection, neuroprotection, *etc*. Furthermore, several studies have reported antibacterial, antifungal, and antiplasmodial activities of FX. FX can be easily isolated from algae, and has no toxicity but it has lower bioavailability, which can be improved. As we have seen, FX also has a positive impact on gut microbiota; technologies to develop its stability can make it available as an important ingredient of pharmaceutical industries replacing synthetic drugs. Better isolation of fucoxanthin and its systemic modification as well as in-depth experiments can reveal and improve its health benefits in future. Being a non-cytotoxic compound, it can open new directions in therapeutic procedures. If the detailed *in vivo* mechanisms of FX are revealed, the multidimensional use of this carotenoid will be possible with guaranteed biosafety. Despite having huge beneficial effects, it has a few clinical trials on human subjects. Therefore, more studies are needed for a better understanding of FX mechanisms inducing different health benefits in humans and other animals.

## References

[B1] Abou M. (2013). Antibacterial Activity of Some Marine Algal Extracts against Most Nosocomial Bacterial Infections. Egypt. J. Exp. Biol. (Bot.). 9 (2), 281–286.

[B2] Afolayan A. F. Bolton J. J. Lategan C. A. Smith P. J. Beukes D. R. (2008). Fucoxanthin, Tetraprenylated Toluquinone and Toluhydroquinone Metabolites from Sargassum Heterophyllum Inhibit the In Vitro Growth of the Malaria Parasite Plasmodium Falciparum. Z Naturforsch C J. Biosci. 63 (11-12), 848–852. 10.1515/znc-2008-11-1211 19227833

[B3] Airanthi M. K. Hosokawa M. Miyashita K. (2011a). Comparative Antioxidant Activity of Edible Japanese Brown Seaweeds. J. Food Sci. 76 (1), C104–C111. 10.1111/j.1750-3841.2010.01915.x 21535637

[B4] Airanthi M. K. Sasaki N. Iwasaki S. Baba N. Abe M. Hosokawa M. (2011b). Effect of Brown Seaweed Lipids on Fatty Acid Composition and Lipid Hydroperoxide Levels of Mouse Liver. J. Agric. Food Chem. 59 (8), 4156–4163. 10.1021/jf104643b 21405010

[B5] Albuquerque D. Nóbrega C. Manco L. Padez C. (2017). The Contribution of Genetics and Environment to Obesity. Br. Med. Bull. 123 (1), 159–173. 10.1093/bmb/ldx022 28910990

[B6] Almeida T. P. Ferreira J. Vettorazzi A. Azqueta A. Rocha E. Ramos A. A. (2018). Cytotoxic Activity of Fucoxanthin, Alone and in Combination with the Cancer Drugs Imatinib and Doxorubicin, in CML Cell Lines. Environ. Toxicol. Pharmacol. 59, 24–33. 10.1016/j.etap.2018.02.006 29518678

[B7] Alok A. Singh I. D. Singh S. Kishore M. Jha P. C. Iqubal M. A. (2017). Probiotics: A New Era of Biotherapy. Adv. Biomed. Res. 6, 31. 10.4103/2277-9175.192625 28401078 PMC5360003

[B8] Andrié Haugan J. Liaaen-Jensen S. (1992). Naturally Occurring Stereoisomers of Fucoxanthin. Phytochemistry 31 (4), 1359–1361. 10.1016/0031-9422(92)80290-u

[B9] Angelakis E. Armougom F. Million M. Raoult D. (2012). The Relationship between Gut Microbiota and Weight Gain in Humans. Future Microbiol. 7 (1), 91–109. 10.2217/fmb.11.142 22191449

[B10] Angelis I. D. Turco L. (2011). Caco-2 Cells as a Model for Intestinal Absorption. Curr. Protoc. Toxicol. Chapter 20 (1), Unit20–6. 10.1002/0471140856.tx2006s47 21400683

[B11] Aronoff S. (1950). The Absorption Spectra of Chlorophyll and Related Compounds. Chem. Rev. 47 (2), 175–195. 10.1021/cr60147a001 24538875

[B12] Asai A. Sugawara T. Ono H. Nagao A. (2004). Biotransformation of Fucoxanthinol into Amarouciaxanthin A in Mice and HepG2 Cells: Formation and Cytotoxicity of Fucoxanthin Metabolites. Drug Metab. Dispos. 32 (2), 205–211. 10.1124/dmd.32.2.205 14744942

[B13] Asai A. Yonekura L. Nagao A. (2008). Low Bioavailability of Dietary Epoxyxanthophylls in Humans. Br. J. Nutr. 100 (2), 273–277. 10.1017/S0007114507895468 18186952

[B14] Ávila-Román J. García-Gil S. Rodríguez-Luna A. Motilva V. Talero E. (2021). Anti-Inflammatory and Anticancer Effects of Microalgal Carotenoids. Mar. Drugs 19 (10), 531. https://www.mdpi.com/1660-3397/19/10/531. 10.3390/md19100531 34677429 PMC8539290

[B15] Berry C. E. Hare J. M. (2004). Xanthine Oxidoreductase and Cardiovascular Disease: Molecular Mechanisms and Pathophysiological Implications. J. Physiol. 555 (Pt 3), 589–606. 10.1113/jphysiol.2003.055913 14694147 PMC1664875

[B16] Berthon J. Y. Nachat-Kappes R. Bey M. Cadoret J. P. Renimel I. Filaire E. (2017). Marine Algae as Attractive Source to Skin Care. Free Radic. Res. 51 (6), 555–567. 10.1080/10715762.2017.1355550 28770671

[B17] Bo T. Shao S. Wu D. Niu S. Zhao J. Gao L. (2017). Relative Variations of Gut Microbiota in Disordered Cholesterol Metabolism Caused by High-Cholesterol Diet and Host Genetics. Microbiologyopen 6 (4), e00491. 10.1002/mbo3.491 PMC5552918 28660729

[B18] Bolhassani A. (2015). Cancer Chemoprevention by Natural Carotenoids as an Efficient Strategy. Anticancer Agents Med. Chem. 15 (8), 1026–1031. https://www.ingentaconnect.com/content/ben/acamc/2015/00000015/00000008/art00011. 10.2174/1871520615666150302125707 25731177

[B19] Bose T. Diedrichs-Möhring M. Wildner G. (2016). Dry Eye Disease and Uveitis: A Closer Look at Immune Mechanisms in Animal Models of Two Ocular Autoimmune Diseases. Autoimmun. Rev. 15 (12), 1181–1192. 10.1016/j.autrev.2016.09.001 27639836

[B20] Bray F. Ferlay J. Soerjomataram I. Siegel R. L. Torre L. A. Jemal A. (2018). Global Cancer Statistics 2018: GLOBOCAN Estimates of Incidence and Mortality Worldwide for 36 Cancers in 185 Countries. CA Cancer J. Clin. 68 (6), 394–424. 10.3322/caac.21492 30207593

[B21] Britton G. Liaaen-Jensen S. Pfander H. (2008). Carotenoids, 4. Birkhäuser Basel: Springer Science & Business Media. natural functions.

[B22] Broecker F. Klumpp J. Moelling K. (2016). Long-term Microbiota and Virome in a Zürich Patient after Fecal Transplantation against *Clostridium difficile* Infection. Ann. N. Y. Acad. Sci. 1372 (1), 29–41. 10.1111/nyas.13100 27286042

[B23] Brown J. M. Attardi L. D. (2005). The Role of Apoptosis in Cancer Development and Treatment Response. Nat. Rev. Cancer 5 (3), 231–237. 10.1038/nrc1560 15738985

[B24] Brunt E. G. Burgess J. G. (2018). The Promise of Marine Molecules as Cosmetic Active Ingredients. Int. J. Cosmet. Sci. 40 (1), 1–15. 10.1111/ics.12435 29057483

[B25] Carmeliet P. (2003). Angiogenesis in Health and Disease. Nat. Med. 9 (6), 653–660. 10.1038/nm0603-653 12778163

[B26] Chang C. J. Lin C. S. Lu C. C. Martel J. Ko Y. F. Ojcius D. M. (2015). Ganoderma Lucidum Reduces Obesity in Mice by Modulating the Composition of the Gut Microbiota. Nat. Commun. 6 (1), 7489. 10.1038/ncomms8489 26102296 PMC4557287

[B27] Chang J. H. Wakefield D. (2002). Uveitis: a Global Perspective. Ocul. Immunol. Inflamm. 10 (4), 263–279. 10.1076/ocii.10.4.263.15592 12854035

[B28] Chang Y. H. Chen Y. L. Huang W. C. Liou C. J. (2018). Fucoxanthin Attenuates Fatty Acid-Induced Lipid Accumulation in FL83B Hepatocytes through Regulated Sirt1/AMPK Signaling Pathway. Biochem. Biophys. Res. Commun. 495 (1), 197–203. 10.1016/j.bbrc.2017.11.022 29113798

[B29] Chen L. Deng H. Cui H. Fang J. Zuo Z. Deng J. (2018). Inflammatory Responses and Inflammation-Associated Diseases in Organs. Oncotarget 9 (6), 7204–7218. 10.18632/oncotarget.23208 29467962 PMC5805548

[B30] Cheng R. Y. Li M. Li S. S. He M. Yu X. H. Shi L. (2017). Vancomycin and Ceftriaxone Can Damage Intestinal Microbiota and Affect the Development of the Intestinal Tract and Immune System to Different Degrees in Neonatal Mice. Pathog. Dis. 75 (8), 1–9. 10.1093/femspd/ftx104 28957452

[B31] Cherkaoui-Malki M. Surapureddi S. El-Hajj H. I. Vamecq J. Andreoletti P. (2012). Hepatic Steatosis and Peroxisomal Fatty Acid Beta-Oxidation. Curr. Drug Metab. 13 (10), 1412–1421. 10.2174/138920012803762765 22978396

[B32] Chitchumroonchokchai C. Failla M. L. (2006). Hydrolysis of Zeaxanthin Esters by Carboxyl Ester Lipase during Digestion Facilitates Micellarization and Uptake of the Xanthophyll by Caco-2 Human Intestinal Cells. J. Nutr. 136 (3), 588–594. 10.1093/jn/136.3.588 16484529

[B33] Cho H. Y. Jedlicka A. E. Reddy S. P. Kensler T. W. Yamamoto M. Zhang L. Y. (2002). Role of NRF2 in Protection against Hyperoxic Lung Injury in Mice. Am. J. Respir. Cell. Mol. Biol. 26 (2), 175–182. 10.1165/ajrcmb.26.2.4501 11804867

[B34] Choi J.-H. Kim N.-H. Kim S.-J. Lee H.-J. Kim S. (2016). Fucoxanthin Inhibits the Inflammation Response in Paw Edema Model through Suppressing MAPKs, Akt, and NFκB. J. Biochem. Mol. Toxicol. 30 (3), 111–119. 10.1002/jbt.21769 26418808

[B35] Chung T. W. Choi H. J. Lee J. Y. Jeong H. S. Kim C. H. Joo M. (2013). Marine Algal Fucoxanthin Inhibits the Metastatic Potential of Cancer Cells. Biochem. Biophys. Res. Commun. 439 (4), 580–585. 10.1016/j.bbrc.2013.09.019 24036125

[B36] Colpitts S. L. Kasper E. J. Keever A. Liljenberg C. Kirby T. Magori K. (2017). A Bidirectional Association between the Gut Microbiota and CNS Disease in a Biphasic Murine Model of Multiple Sclerosis. Gut Microbes 8 (6), 561–573. 10.1080/19490976.2017.1353843 28708466 PMC5730387

[B37] Cook M. D. Allen J. M. Pence B. D. Wallig M. A. Gaskins H. R. White B. A. (2016). Exercise and Gut Immune Function: Evidence of Alterations in Colon Immune Cell Homeostasis and Microbiome Characteristics with Exercise Training. Immunol. Cell. Biol. 94 (2), 158–163. 10.1038/icb.2015.108 26626721

[B38] Cowman A. F. Healer J. Marapana D. Marsh K. (2016). Malaria: Biology and Disease. Cell. 167 (3), 610–624. 10.1016/j.cell.2016.07.055 27768886

[B39] Cox S. Abu-Ghannam N. Gupta S. (2010). An Assessment of the Antioxidant and Antimicrobial Activity of Six Species of Edible. Ir. seaweeds 17 (1), 205–220. 10.21427/D7HC92

[B40] Dai Y.-L. Jiang Y.-F. Lu Y.-A. Yu J.-B. Kang M.-C. Jeon Y.-J. (2021). Fucoxanthin-rich Fraction from Sargassum Fusiformis Alleviates Particulate Matter-Induced Inflammation In Vitro and In Vivo. Toxicol. Rep. 8, 349–358. 10.1016/j.toxrep.2021.02.005 33665132 PMC7898073

[B41] Damodharan K. Palaniyandi S. A. Yang S. H. Suh J. W. (2016). Functional Probiotic Characterization and In Vivo Cholesterol-Lowering Activity of Lactobacillus Helveticus Isolated from Fermented Cow Milk. J. Microbiol. Biotechnol. 26 (10), 1675–1686. 10.4014/jmb.1603.03005 27435541

[B42] Das S. K. Hashimoto T. Kanazawa K. (2008). Growth Inhibition of Human Hepatic Carcinoma HepG2 Cells by Fucoxanthin Is Associated with Down-Regulation of Cyclin D. Biochim. Biophys. Acta 1780 (4), 743–749. 10.1016/j.bbagen.2008.01.003 18230364

[B43] Das S. K. Hashimoto T. Shimizu K. Yoshida T. Sakai T. Sowa Y. (2005). Fucoxanthin Induces Cell Cycle Arrest at G0/G1 Phase in Human Colon Carcinoma Cells through Up-Regulation of p21WAF1/Cip1. Biochim. Biophys. Acta 1726 (3), 328–335. 10.1016/j.bbagen.2005.09.007 16236452

[B44] Delbrut A. Albina P. Lapierre T. Pradelles R. Dubreucq E. (2018). Fucoxanthin and Polyunsaturated Fatty Acids Co-extraction by a Green Process. Molecules 23 (4), 874. 10.3390/molecules23040874 PMC6017215 29641444

[B45] Delzenne N. M. Cani P. D. (2011). Interaction between Obesity and the Gut Microbiota: Relevance in Nutrition. Annu. Rev. Nutr. 31, 15–31. 10.1146/annurev-nutr-072610-145146 21568707

[B46] Dembitsky V. M. Maoka T. (2007). Allenic and Cumulenic Lipids. Prog. Lipid Res. 46 (6), 328–375. 10.1016/j.plipres.2007.07.001 17765976

[B47] Devine D. A. Hancock R. E. (2002). Cationic Peptides: Distribution and Mechanisms of Resistance. Curr. Pharm. Des. 8 (9), 703–714. 10.2174/1381612023395501 11945166

[B48] Ding Y. F. Wu Z. H. Wei Y. J. Shu L. Peng Y. R. (2017). Hepatic Inflammation-Fibrosis-Cancer axis in the Rat Hepatocellular Carcinoma Induced by Diethylnitrosamine. J. Cancer Res. Clin. Oncol. 143 (5), 821–834. 10.1007/s00432-017-2364-z 28238064 PMC11819159

[B49] D’Orazio N. Gemello E. Gammone M. A. De Girolamo M. Ficoneri C. Riccioni G. (2012). Fucoxantin: A Treasure from the Sea. Mar. Drugs. 10 (3), 604–616. https://www.mdpi.com/1660-3397/10/3/604. 10.3390/md10030604 22611357 PMC3347018

[B50] Dunbar E. Yachnis A. T. (2010). Glioma Diagnosis: Immunohistochemistry and beyond. Adv. Anat. Pathol. 17 (3), 187–201. 10.1097/PAP.0b013e3181d98cd9 20418673

[B51] Duncan F. J. Martin J. R. Wulff B. C. Stoner G. D. Tober K. L. Oberyszyn T. M. (2009). Topical Treatment with Black Raspberry Extract Reduces Cutaneous UVB-Induced Carcinogenesis and Inflammation. Cancer Prev. Res. (Phila) 2 (7), 665–672. 10.1158/1940-6207.CAPR-08-0193 19584078 PMC3874934

[B52] Durand C. P. Andalib M. Dunton G. F. Wolch J. Pentz M. A. (2011). A Systematic Review of Built Environment Factors Related to Physical Activity and Obesity Risk: Implications for Smart Growth Urban Planning. Obes. Rev. 12 (5), e173–82. 10.1111/j.1467-789X.2010.00826.x 21348918 PMC3079793

[B53] During A. Harrison E. H. (2004). Intestinal Absorption and Metabolism of Carotenoids: Insights from Cell Culture. Arch. Biochem. Biophys. 430 (1), 77–88. 10.1016/j.abb.2004.03.024 15325914

[B54] Englert G. Bjørnland T. Liaaen-Jensen S. (1990). 1D and 2D NMR Study of Some Allenic Carotenoids of the Fucoxanthin Series. Magn. Reson. Chem. 28 (6), 519–528. 10.1002/mrc.1260280610

[B55] Esselstyn C. B. (2007). Prevent and Reverse Heart Disease: The Revolutionary, Scientifically Proven, Nutrition-Based Cure. New York, NY: Penguin.

[B56] Evan G. I. Vousden K. H. (2001). Proliferation, Cell Cycle and Apoptosis in Cancer. Nature 411 (6835), 342–348. 10.1038/35077213 11357141

[B57] Fabbrini E. Sullivan S. Klein S. (2010). Obesity and Nonalcoholic Fatty Liver Disease: Biochemical, Metabolic, and Clinical Implications. Hepatology 51 (2), 679–689. 10.1002/hep.23280 20041406 PMC3575093

[B58] Fernández-García E. Carvajal-Lérida I. Jarén-Galán M. Garrido-Fernández J. Pérez-Gálvez A. Hornero-Méndez D. (2012). Carotenoids Bioavailability from Foods: From Plant Pigments to Efficient Biological Activities. Food Res. Int. 46 (2), 438–450. 10.1016/j.foodres.2011.06.007

[B59] Foo S. C. Yusoff F. M. Imam M. U. Foo J. B. Ismail N. Azmi N. H. (2019). Increased Fucoxanthin in Chaetoceros Calcitrans Extract Exacerbates Apoptosis in Liver Cancer Cells via Multiple Targeted Cellular Pathways. Biotechnol. Rep. (Amst) 21, e00296. 10.1016/j.btre.2018.e00296 30581767 PMC6296166

[B60] Foster M. T. Gentile C. L. Cox-York K. Wei Y. Wang D. Estrada A. L. (2016). Fuzhuan Tea Consumption Imparts Hepatoprotective Effects and Alters Intestinal Microbiota in High Saturated Fat Diet-Fed Rats. Mol. Nutr. Food Res. 60 (5), 1213–1220. 10.1002/mnfr.201500654 26890069

[B61] Galasso C. Corinaldesi C. Sansone C. (2017). Carotenoids from Marine Organisms: Biological Functions and Industrial Applications. Antioxidants (Basel) 6 (4), 96. 10.3390/antiox6040096 PMC5745506 29168774

[B62] Gammone M. A. D'Orazio N. (2015). Anti-obesity Activity of the Marine Carotenoid Fucoxanthin. Mar. Drugs 13 (4), 2196–2214. 10.3390/md13042196 25871295 PMC4413207

[B63] Gammone M. A. Gemello E. Riccioni G. D'orazio N. (2014). Marine Bioactives and Potential Application in Sports. Mar. Drugs 12 (5), 2357–2382. 10.3390/md12052357 24796298 PMC4052294

[B64] Gao F. Teles Cabanelas Itd I. Wijffels R. H. Barbosa M. J. (2020). Process Optimization of Fucoxanthin Production with Tisochrysis Lutea. Bioresour. Technol. 315, 123894. 10.1016/j.biortech.2020.123894 32736321

[B65] Gill S. S. Tuteja N. (2010). Reactive Oxygen Species and Antioxidant Machinery in Abiotic Stress Tolerance in Crop Plants. Plant Physiol. Biochem. 48 (12), 909–930. 10.1016/j.plaphy.2010.08.016 20870416

[B66] Gille A. Stojnic B. Derwenskus F. Trautmann A. Schmid-Staiger U. Posten C. (2019). A Lipophilic Fucoxanthin-Rich Phaeodactylum Tricornutum Extract Ameliorates Effects of Diet-Induced Obesity in C57BL/6J Mice. Nutrients 11 (4), 796. 10.3390/nu11040796 PMC6521120 30959933

[B67] Goldstein D. E. Little R. R. Lorenz R. A. Malone J. I. Nathan D. Peterson C. M. (2004). Tests of Glycemia in Diabetes. Tests glycemia diabetes 27 (7), 1761–1773. 10.2337/diacare.27.7.1761 15220264

[B68] Grasa-López A. Miliar-García Á. Quevedo-Corona L. Paniagua-Castro N. Escalona-Cardoso G. Reyes-Maldonado E. (2016). Undaria Pinnatifida and Fucoxanthin Ameliorate Lipogenesis and Markers of Both Inflammation and Cardiovascular Dysfunction in an Animal Model of Diet-Induced Obesity. Mar. Drugs 14 (8), 148. 10.3390/md14080148 PMC4999909 27527189

[B69] Gundermann K. Büchel C. (2014). “Structure and Functional Heterogeneity of Fucoxanthin-Chlorophyll Proteins in Diatoms,” in The Structural Basis of Biological Energy Generation (New York, NY: Springer). 10.1007/978-94-017-8742-0_2

[B70] Guo B. Oliviero T. Fogliano V. Ma Y. Chen F. Capuano E. (2020). Gastrointestinal Bioaccessibility and Colonic Fermentation of Fucoxanthin from the Extract of the Microalga Nitzschia Laevis. J. Agric. Food Chem. 68 (7), 1844–1850. 10.1021/acs.jafc.9b02496 31081326 PMC7034079

[B71] Guo L. Dang M. Song Q. Zhang W. Li B. (2020). Protective Effect of Fucoxanthin on Ovariectomy-Induced Osteoporosis in Rats. Phcog. Mag. 16 (69), 242. 10.4103/pm.pm_340_19

[B72] Ha A. W. Na S. J. Kim W. K. (2013). Antioxidant Effects of Fucoxanthin Rich Powder in Rats Fed with High Fat Diet. Nutr. Res. Pract. 7 (6), 475–480. 10.4162/nrp.2013.7.6.475 24353833 PMC3865270

[B73] Ha Y.-J. Choi Y. S. Oh Y. R. Kang E. H. Khang G. Park Y.-B. (2021). Fucoxanthin Suppresses Osteoclastogenesis via Modulation of MAP Kinase and Nrf2 Signaling. Mar. Drugs 19 (3), 132. 10.3390/md19030132 33673704 PMC7997314

[B74] Hardie D. G. Pan D. A. (2002). Regulation of Fatty Acid Synthesis and Oxidation by the AMP-Activated Protein Kinase. Biochem. Soc. Trans. 30 (Pt 6), 1064–1070. 10.1042/bst0301064 12440973

[B75] Harding A. H. Sargeant L. A. Welch A. Oakes S. Luben R. N. Bingham S. (2001). Fat Consumption and HbA(1c) Levels: the EPIC-Norfolk Study. Diabetes Care 24 (11), 1911–1916. 10.2337/diacare.24.11.1911 11679456

[B76] Hashimoto T. Ozaki Y. Mizuno M. Yoshida M. Nishitani Y. Azuma T. (2012). Pharmacokinetics of Fucoxanthinol in Human Plasma after the Oral Administration of Kombu Extract. Br. J. Nutr. 107 (11), 1566–1569. 10.1017/S0007114511004879 21920061

[B77] Heo S.-J. Ko S.-C. Kang S.-M. Kang H.-S. Kim J.-P. Kim S.-H. (2008). Cytoprotective Effect of Fucoxanthin Isolated from Brown Algae Sargassum Siliquastrum against H2O2-Induced Cell Damage. Eur. Food Res. Technol. 228 (1), 145–151. 10.1007/s00217-008-0918-7

[B78] Heo S. J. Jeon Y. J. (2009). Protective Effect of Fucoxanthin Isolated from Sargassum Siliquastrum on UV-B Induced Cell Damage. J. Photochem Photobiol. B 95 (2), 101–107. 10.1016/j.jphotobiol.2008.11.011 19264501

[B79] Heo S. J. Yoon W. J. Kim K. N. Ahn G. N. Kang S. M. Kang D. H. (2010). Evaluation of Anti-inflammatory Effect of Fucoxanthin Isolated from Brown Algae in Lipopolysaccharide-Stimulated RAW 264.7 Macrophages. Food Chem. Toxicol. 48 (8-9), 2045–2051. 10.1016/j.fct.2010.05.003 20457205

[B80] Heo S. J. Yoon W. J. Kim K. N. Oh C. Choi Y. U. Yoon K. T. (2012). Anti-inflammatory Effect of Fucoxanthin Derivatives Isolated from Sargassum Siliquastrum in Lipopolysaccharide-Stimulated RAW 264.7 Macrophage. Food Chem. Toxicol. 50 (9), 3336–3342. 10.1016/j.fct.2012.06.025 22735499

[B81] Hidalgo I. J. Raub T. J. Borchardt R. T. (2011). Characterization of the Human Colon Carcinoma Cell Line (Caco-2) as a Model System for Intestinal Epithelial Permeability. Gastroenterology 96, 736–749. 2914637

[B82] Hii S. Choong P. Woo K. Wong C. (2010). Stab. Stud. fucoxanthin Sargassum Bind. 4 (10), 4580–4584.

[B83] Hitoe S. Shimoda H. (2017). Seaweed Fucoxanthin Supplementation Improves Obesity Parameters in Mild Obese Japanese Subjects. Ffhd 7 (4), 246–262. 10.31989/ffhd.v7i4.333

[B84] Hosokawa M. Kudo M. Maeda H. Kohno H. Tanaka T. Miyashita K. (2004). Fucoxanthin Induces Apoptosis and Enhances the Antiproliferative Effect of the PPARgamma Ligand, Troglitazone, on Colon Cancer Cells. Biochim. Biophys. Acta 1675 (1-3), 113–119. 10.1016/j.bbagen.2004.08.012 15535974

[B85] Hosokawa M. Miyashita T. Nishikawa S. Emi S. Tsukui T. Beppu F. (2010). Fucoxanthin Regulates Adipocytokine mRNA Expression in White Adipose Tissue of Diabetic/obese KK-Ay Mice. Arch. Biochem. Biophys. 504 (1), 17–25. 10.1016/j.abb.2010.05.031 20515643

[B86] Hosokawa M. Okada T. Mikami N. Konishi I. Miyashita K. (2009). Bio-functions of Marine Carotenoids. Food Sci. Biotechnol. 18 (1), 1–11.

[B87] Hosokawa M. Wanezaki S. Miyauchi K. Kurihara H. Kohno H. Kawabata J. (1999). Apoptosis-Inducing Effect of Fucoxanthin on Human Leukemia Cell Line HL-60. Fstr 5 (3), 243–246. line HL-60. 10.3136/fstr.5.243

[B88] Hou L.-L. Xu Q.-J. Hu G.-Q. Xie S.-Q. (2014). Fucoxanthin Induces Human MCF-7 Breast Cancer Cells Apoptosis via Endoplasmic Reticulum Pathway. J. Chin. Pharm. Sci. 49 (2), 117–120. 10.11669/cpj.2014.02.007

[B89] Hu L. Chen W. Tian F. Yuan C. Wang H. Yue H. (2018). Neuroprotective Role of Fucoxanthin against Cerebral Ischemic/reperfusion Injury through Activation of Nrf2/HO-1 Signaling. Biomed. Pharmacother. 106, 1484–1489. 10.1016/j.biopha.2018.07.088 30119223

[B90] Hu X. Li Y. Li C. Fu Y. Cai F. Chen Q. (2012). Combination of Fucoxanthin and Conjugated Linoleic Acid Attenuates Body Weight Gain and Improves Lipid Metabolism in High-Fat Diet-Induced Obese Rats. Arch. Biochem. Biophys. 519 (1), 59–65. 10.1016/j.abb.2012.01.011 22289788

[B91] Ichihara S. Yamada Y. (2008). Genetic Factors for Human Obesity. Cell. Mol. Life Sci. 65 (7-8), 1086–1098. 10.1007/s00018-007-7453-8 18097636 PMC11131721

[B92] Iio K. Okada Y. Ishikura M. (2011a). Bacterial Reverse Mutation Test and Micronucleus Test of Fucoxanthin Oil from Microalgae. Shokuhin Eiseigaku Zasshi 52 (3), 190–193. 10.3358/shokueishi.52.190 21720125

[B93] Iio K. Okada Y. Ishikura M. (2011b). Single and 13-week Oral Toxicity Study of Fucoxanthin Oil from Microalgae in Rats. Shokuhin Eiseigaku Zasshi 52 (3), 183–189. 10.3358/shokueishi.52.183 21720124

[B94] Ikeda K. Kitamura A. Machida H. Watanabe M. Negishi H. Hiraoka J. (2003). Effect of Undaria Pinnatifida (Wakame) on the Development of Cerebrovascular Diseases in Stroke-Prone Spontaneously Hypertensive Rats. Clin. Exp. Pharmacol. Physiol. 30 (1-2), 44–48. 10.1046/j.1440-1681.2003.03786.x 12542452

[B95] Ishikawa C. Tafuku S. Kadekaru T. Sawada S. Tomita M. Okudaira T. (2008). Anti-adult T-Cell Leukemia Effects of Brown Algae Fucoxanthin and its Deacetylated Product, Fucoxanthinol. Int. J. Cancer 123 (11), 2702–2712. 10.1002/ijc.23860 18798263

[B96] Ito K. Carracedo A. Weiss D. Arai F. Ala U. Avigan D. E. (2012). A PML–PPAR-δ Pathway for Fatty Acid Oxidation Regulates Hematopoietic Stem Cell Maintenance. Nat. Med. 18 (9), 1350–1358. 10.1038/nm.2882 22902876 PMC3566224

[B97] Jang W. S. Choung S. Y. (2013). Antiobesity Effects of the Ethanol Extract ofLaminaria japonicaAreshoung in High-Fat-Diet-Induced Obese Rat. Evidence-Based Complementary Altern. Med. 2013, 492807. 10.1155/2013/492807 PMC3556441 23365609

[B98] Jastroch M. Ussar S. Keipert S. (2020). Gut Microbes Controlling Blood Sugar: No Fire Required!. Cell. Metab. 31 (3), 443–444. 10.1016/j.cmet.2020.02.007 32130877

[B99] Jeon S. M. Kim H. J. Woo M. N. Lee M. K. Shin Y. C. Park Y. B. (2010). Fucoxanthin-rich Seaweed Extract Suppresses Body Weight Gain and Improves Lipid Metabolism in High-Fat-Fed C57BL/6J Mice. Biotechnol. J. 5 (9), 961–969. 10.1002/biot.201000215 20845386

[B100] Jia S. Gao Z. Yan S. Chen Y. Sun C. Li X. (2016). Anti-Obesity and Hypoglycemic Effects of Poncirus Trifoliata L. Extracts in High-Fat Diet C57BL/6 Mice. Molecules 21 (4), 453. 10.3390/molecules21040453 27058520 PMC6273343

[B101] Jilka R. L. (2013). The Relevance of Mouse Models for Investigating Age-Related Bone Loss in Humans. J. Gerontol. A Biol. Sci. Med. Sci. 68 (10), 1209–1217. 10.1093/gerona/glt046 23689830 PMC3779631

[B102] Jiménez‐Escrig A. Jiménez‐Jiménez I. Pulido R. Saura‐Calixto F. J. (2001). Antioxidant Activity of Fresh and Processed Edible Seaweeds. J. Sci. Food Agric. 81 (5), 530–534. 10.1002/jsfa.842

[B103] Jin X. Zhao T. Shi D. Ye M. B. Yi Q. (2019). Protective Role of Fucoxanthin in Diethylnitrosamine-Induced Hepatocarcinogenesis in Experimental Adult Rats. Drug Dev. Res. 80 (2), 209–217. 10.1002/ddr.21451 30379338

[B104] Jin Y. Qiu S. Shao N. Zheng J. (2018). Fucoxanthin and Tumor Necrosis Factor-Related Apoptosis-Inducing Ligand (TRAIL) Synergistically Promotes Apoptosis of Human Cervical Cancer Cells by Targeting PI3K/Akt/NF-Κb Signaling Pathway. Med. Sci. Monit. 24, 11–18. 10.12659/msm.905360 29291370 PMC5759513

[B105] Jounai N. Kobiyama K. Takeshita F. Ishii K. J. (2012). Recognition of Damage-Associated Molecular Patterns Related to Nucleic Acids during Inflammation and Vaccination. Front. Cell. Infect. Microbiol. 2, 168. 10.3389/fcimb.2012.00168 23316484 PMC3539075

[B106] Jung H. A. Ali M. Y. Choi R. J. Jeong H. O. Chung H. Y. Choi J. S. (2016). Kinetics and Molecular Docking Studies of Fucosterol and Fucoxanthin, BACE1 Inhibitors from Brown Algae Undaria Pinnatifida and Ecklonia Stolonifera. Food Chem. Toxicol. 89, 104–111. 10.1016/j.fct.2016.01.014 26825629

[B107] Jung H. A. Islam M. N. Lee C. M. Jeong H. O. Chung H. Y. Woo H. C. (2012). Promising Antidiabetic Potential of Fucoxanthin Isolated from the Edible Brown Algae *Eisenia bicyclis* and Undaria Pinnatifida. Fish. Sci. 78 (6), 1321–1329. 10.1007/s12562-012-0552-y

[B108] Kanda T. Nishida A. Ohno M. Imaeda H. Shimada T. Inatomi O. (2016). Enterococcus Durans TN-3 Induces Regulatory T Cells and Suppresses the Development of Dextran Sulfate Sodium (DSS)-Induced Experimental Colitis. PLoS One 11 (7), e0159705. 10.1371/journal.pone.0159705 27438072 PMC4954729

[B109] Kang S. I. Ko H. C. Shin H. S. Kim H. M. Hong Y. S. Lee N. H. (2011). Fucoxanthin Exerts Differing Effects on 3T3-L1 Cells According to Differentiation Stage and Inhibits Glucose Uptake in Mature Adipocytes. Biochem. Biophys. Res. Commun. 409 (4), 769–774. 10.1016/j.bbrc.2011.05.086 21621511

[B110] Karpiński T. M. Adamczak A. J. A. (2019). Fucoxanthin-An Antibacterial Carotenoid. Antioxidants (Basel). 8 (8), 239. 10.3390/antiox8080239 PMC6720875 31344844

[B111] Kawee-Ai A. Kuntiya A. Kim S. M. (2013). Anticholinesterase and Antioxidant Activities of Fucoxanthin Purified from the Microalga Phaeodactylum Tricornutum. Nat. Prod. Commun. 8 (10), 1381–1386. https://www.ncbi.nlm.nih.gov/pubmed/24354180. 10.1177/1934578x1300801010 24354180

[B112] Khachik F. Beecher G. R. Goli M. B. Lusby W. R. Smith J. C. Jr (1992). Separation and Identification of Carotenoids and Their Oxidation Products in the Extracts of Human Plasma. Anal. Chem. 64 (18), 2111–2122. 10.1021/ac00042a016 1416048

[B113] Khan A. A. Morrison A. Hanley D. A. Felsenberg D. Mccauley L. K. O'ryan F. (2015). Diagnosis and Management of Osteonecrosis of the Jaw: a Systematic Review and International Consensus. J. Bone Min. Res. 30 (1), 3–23. 10.1002/jbmr.2405 25414052

[B114] Khan M. N. Choi J. S. Lee M. C. Kim E. Nam T. J. Fujii H. (2008a). Anti-inflammatory Activities of Methanol Extracts from Various Seaweed Species. J. Environ. Biol. 29 (4), 465–469. 19195382

[B115] Khan M. N. Lee M. C. Kang J. Y. Park N. G. Fujii H. Hong Y. K. (2008b). Effects of the Brown Seaweed Undaria Pinnatifida on Erythematous Inflammation Assessed Using Digital Photo Analysis. Phytother. Res. 22 (5), 634–639. 10.1002/ptr.2349 18384198

[B116] Khan S. B. Kong C.-S. Kim J.-A. Kim S.-K. (2010). Protective Effect of Amphiroa Dilatata on ROS Induced Oxidative Damage and MMP Expressions in HT1080 Cells. Biotechnol. Bioproc E 15 (1), 191–198. 10.1007/s12257-009-0052-9

[B117] Khosla S. (2013). Pathogenesis of Age-Related Bone Loss in Humans. J. Gerontol. A Biol. Sci. Med. Sci. 68 (10), 1226–1235. 10.1093/gerona/gls163 22923429 PMC3826857

[B118] Khosla S. Riggs B. L. (2005). Pathophysiology of Age-Related Bone Loss and Osteoporosis. Endocrinol. Metab. Clin. North Am. 34 (4), 1015–xi. 10.1016/j.ecl.2005.07.009 16310636

[B119] Kim C. W. Moon Y. A. Park S. W. Cheng D. Kwon H. J. Horton J. D. (2010a). Induced Polymerization of Mammalian Acetyl-CoA Carboxylase by MIG12 Provides a Tertiary Level of Regulation of Fatty Acid Synthesis. Proc. Natl. Acad. Sci. U. S. A. 107 (21), 9626–9631. 10.1073/pnas.1001292107 20457939 PMC2906888

[B120] Kim K. N. Heo S. J. Kang S. M. Ahn G. Jeon Y. J. (2010a). Fucoxanthin Induces Apoptosis in Human Leukemia HL-60 Cells through a ROS-Mediated Bcl-xL Pathway. Toxicol Vitro 24 (6), 1648–1654. 10.1016/j.tiv.2010.05.023 20594983

[B121] Kim K. N. Heo S. J. Yoon W. J. Kang S. M. Ahn G. Yi T. H. (2010b). Fucoxanthin Inhibits the Inflammatory Response by Suppressing the Activation of NF-Κb and MAPKs in Lipopolysaccharide-Induced RAW 264.7 Macrophages. Eur. J. Pharmacol. 649 (1-3), 369–375. 10.1016/j.ejphar.2010.09.032 20868674

[B122] Kim J. M. Araki S. Kim D. J. Park C. B. Takasuka N. Baba-Toriyama H. (1998). Chemopreventive Effects of Carotenoids and Curcumins on Mouse Colon Carcinogenesis after 1,2-dimethylhydrazine Initiation. Carcinogenesis 19 (1), 81–85. 10.1093/carcin/19.1.81 9472697

[B123] Kim S. M. Jung Y. J. Kwon O. N. Cha K. H. Um B. H. Chung D. (2012a). A Potential Commercial Source of Fucoxanthin Extracted from the Microalga Phaeodactylum Tricornutum. Appl. Biochem. Biotechnol. 166 (7), 1843–1855. 10.1007/s12010-012-9602-2 22371063

[B124] Kim S. M. Kang S.-W. Kwon O.-N. Chung D. Pan C.-H. (2012b). Fucoxanthin as a Major Carotenoid in Isochrysis Aff. Galbana: Characterization of Extraction for Commercial Application. J. Korean Soc. Appl. Biol. Chem. 55 (4), 477–483. 10.1007/s13765-012-2108-3

[B125] Kirk S. Frank J. A. Karlik S. (2004). Angiogenesis in Multiple Sclerosis: Is it Good, Bad or an Epiphenomenon? J. Neurol. Sci. 217 (2), 125–130. 10.1016/j.jns.2003.10.016 14706213

[B126] Kitagawa Y. Bujo H. Takahashi K. Shibasaki M. Ishikawa K. Yagui K. (2004). Impaired Glucose Tolerance Is Accompanied by Decreased Insulin Sensitivity in Tissues of Mice Implanted with Cells that Overexpress Resistin. Diabetologia 47 (10), 1847–1853. 10.1007/s00125-004-1530-4 15502922

[B127] Koo S. Y. Hwang J. H. Yang S. H. Um J. I. Hong K. W. Kang K. (2019). Anti-Obesity Effect of Standardized Extract of Microalga Phaeodactylum Tricornutum Containing Fucoxanthin. Mar. Drugs 17 (5), 311. 10.3390/md17050311 PMC6562887 31137922

[B128] Kopelman P. G. (2000). Obesity as a Medical Problem. Nature 404 (6778), 635–643. 10.1038/35007508 10766250

[B129] Kose O. Arabaci T. Yemenoglu H. Kara A. Ozkanlar S. Kayis S. (2016). Influences of Fucoxanthin on Alveolar Bone Resorption in Induced Periodontitis in Rat Molars. Mar. Drugs 14 (4), 70. 10.3390/md14040070 PMC4849074 27043583

[B130] Kotake-Nara E. Asai A. Nagao A. (2005a). Neoxanthin and Fucoxanthin Induce Apoptosis in PC-3 Human Prostate Cancer Cells. Cancer Lett. 220 (1), 75–84. 10.1016/j.canlet.2004.07.048 15737690

[B131] Kotake-Nara E. Kushiro M. Zhang H. Sugawara T. Miyashita K. Nagao A. (2001). Carotenoids Affect Proliferation of Human Prostate Cancer Cells. J. Nutr. 131 (12), 3303–3306. 10.1093/jn/131.12.3303 11739884

[B132] Kotake-Nara E. Terasaki M. Nagao A. (2005c). Characterization of Apoptosis Induced by Fucoxanthin in Human Promyelocytic Leukemia Cells. Biosci. Biotechnol. Biochem. 69 (1), 224–227. 10.1271/bbb.69.224 15665492

[B133] Kotake-Nara E. Sugawara T. Nagao A. (2005b). Antiproliferative Effect of Neoxanthin and Fucoxanthin on Cultured Cells. Fish. Sci. 71 (2), 459–461. 10.1111/j.1444-2906.2005.00986.x

[B134] Krinsky N. I. Mayne S. T. Sies H. (2004). Carotenoids in Health and Disease. Boca Raton, FL: CRC Press.

[B135] Kwak M. K. Itoh K. Yamamoto M. Sutter T. R. Kensler T. W. (2001). Role of Transcription Factor Nrf2 in the Induction of Hepatic Phase 2 and Antioxidative Enzymes In Vivo by the Cancer Chemoprotective Agent, 3H-1, 2-Dimethiole-3-Thione. Mol. Med. 7 (2), 135–145. 3 H-1, 2-dithiole-3-thione. 10.1007/bf03401947 11471548 PMC1950021

[B136] Lavanya R. Veerappan N. (2011). Antibacterial Potential of Six Seaweeds Collected from Gulf of Mannar of Southeast Coast of India. Adv. Biol. Researc 5 (1), 38–44.

[B137] Lecomte V. Kaakoush N. O. Maloney C. A. Raipuria M. Huinao K. D. Mitchell H. M. (2015). Changes in Gut Microbiota in Rats Fed a High Fat Diet Correlate with Obesity-Associated Metabolic Parameters. PLoS One 10 (5), e0126931. 10.1371/journal.pone.0126931 25992554 PMC4436290

[B138] Lee J. M. Grabb M. C. Zipfel G. J. Choi D. W. (2000). Brain Tissue Responses to Ischemia. J. Clin. Invest. 106 (6), 723–731. 10.1172/JCI11003 10995780 PMC381398

[B139] Lee M. J. Wu Y. Fried S. K. (2013). Adipose Tissue Heterogeneity: Implication of Depot Differences in Adipose Tissue for Obesity Complications. Mol. Asp. Med. 34 (1), 1–11. 10.1016/j.mam.2012.10.001 PMC3549425 23068073

[B140] Li D. Zhang Q. Huang L. Chen Z. Zou C. Ma Y. (2021). Fabricating Hydrophilic Particles with Oleic Acid and Bovine Serum Albumin to Improve the Dispersibility and Bioaccessibility of Fucoxanthin in Water. Food Hydrocoll. 118, 106752. 10.1016/j.foodhyd.2021.106752

[B141] Li S. Chen G. Zhang C. Wu M. Wu S. Liu Q. J. F. S. (2014). Research Progress of Natural Antioxidants in Foods for the Treatment of Diseases. Food Sci. Hum. Wellness 3 (3-4), 110–116. 10.1016/j.fshw.2014.11.002

[B142] Lim M. W. S. Tan K. M. Chew L. Y. Kong K. W. Yan S. W. (2018). Application of Two-Level Full Factorial Design for the Extraction of Fucoxanthin and Antioxidant Activities from Sargassum Siliquosum and Sargassum Polycystum. J. Aquatic Food Prod. Technol. 27 (4), 446–463. 10.1080/10498850.2018.1448918

[B143] Lippert K. Kedenko L. Antonielli L. Kedenko I. Gemeier C. Leitner M. (2017). Gut Microbiota Dysbiosis Associated with Glucose Metabolism Disorders and the Metabolic Syndrome in Older Adults. Benef. Microbes 8 (4), 545–556. 10.3920/BM2016.0184 28701081

[B144] Liu C. L. Chiu Y. T. Hu M. L. (2011). Fucoxanthin Enhances HO-1 and NQO1 Expression in Murine Hepatic BNL CL.2 Cells through Activation of the Nrf2/ARE System Partially by its Pro-oxidant Activity. J. Agric. Food Chem. 59 (20), 11344–11351. 10.1021/jf2029785 21919437

[B145] Liu C. L. Huang Y. S. Hosokawa M. Miyashita K. Hu M. L. (2009). Inhibition of Proliferation of a Hepatoma Cell Line by Fucoxanthin in Relation to Cell Cycle Arrest and Enhanced Gap Junctional Intercellular Communication. Chem. Biol. Interact. 182 (2-3), 165–172. 10.1016/j.cbi.2009.08.017 19737546

[B146] Liu J. Yue S. Yang Z. Feng W. Meng X. Wang A. (2018). Oral Hydroxysafflor Yellow A Reduces Obesity in Mice by Modulating the Gut Microbiota and Serum Metabolism. Pharmacol. Res. 134, 40–50. 10.1016/j.phrs.2018.05.012 29787870

[B147] Liu Y. Liu M. Zhang X. Chen Q. Chen H. Sun L. (2016a). Protective Effect of Fucoxanthin Isolated from Laminaria Japonica against Visible Light-Induced Retinal Damage Both In Vitro and In Vivo. J. Agric. Food Chem. 64 (2), 416–424. 10.1021/acs.jafc.5b05436 26708928

[B148] Liu Y. Zheng J. Zhang Y. Wang Z. Yang Y. Bai M. (2016). Fucoxanthin Activates Apoptosis via Inhibition of PI3K/Akt/mTOR Pathway and Suppresses Invasion and Migration by Restriction of P38-MMP-2/9 Pathway in Human Glioblastoma Cells. Neurochem. Res. 41 (10), 2728–2751. 10.1007/s11064-016-1989-7 27394418

[B149] Liu Y. Qiao Z. Liu W. Hou Z. Zhang D. Huang L. (2019). Oleic Acid as a Protein Ligand Improving Intestinal Absorption and Ocular Benefit of Fucoxanthin in Water through Protein-Based Encapsulation. Food Funct. 10 (7), 4381–4395. 10.1039/c9fo00814d 31282516

[B150] Liu Z. Sun X. Sun X. Wang S. Xu Y. (2019). Fucoxanthin Isolated from Undaria Pinnatifida Can Interact with *Escherichia coli* and Lactobacilli in the Intestine and Inhibit the Growth of Pathogenic Bacteria. J. Ocean. Univ. China 18 (4), 926–932. 10.1007/s11802-019-4019-y

[B151] Long Y. Cao X. Zhao R. Gong S. Jin L. Feng C. (2020). Fucoxanthin Treatment Inhibits Nasopharyngeal Carcinoma Cell Proliferation through Induction of Autophagy Mechanism. Environ. Toxicol. 35 (10), 1082–1090. 10.1002/tox.22944 32449842

[B152] Ma Z. Khalid N. Shu G. Zhao Y. Kobayashi I. Neves M. A. (2019). Fucoxanthin-Loaded Oil-In-Water Emulsion-Based Delivery Systems: Effects of Natural Emulsifiers on the Formulation, Stability, and Bioaccessibility. ACS Omega 4 (6), 10502–10509. 10.1021/acsomega.9b00871 31460147 PMC6648090

[B153] MacArtain P. Gill C. I. Brooks M. Campbell R. Rowland I. R. (2007). Nutritional Value of Edible Seaweeds. Nutr. Rev. 65 (12 Pt 1), 535–543. 10.1301/nr.2007.dec.535-543 18236692

[B154] Maeda H. Hosokawa M. Sashima T. Funayama K. Miyashita K. (2007a). Effect of Medium-Chain Triacylglycerols on Anti-obesity Effect of Fucoxanthin. J. Oleo Sci. 56 (12), 615–621. 10.5650/jos.56.615 17992001

[B155] Maeda H. Hosokawa M. Sashima T. Funayama K. Miyashita K. (2005). Fucoxanthin from Edible Seaweed, Undaria Pinnatifida, Shows Antiobesity Effect through UCP1 Expression in White Adipose Tissues. Biochem. Biophys. Res. Commun. 332 (2), 392–397. 10.1016/j.bbrc.2005.05.002 15896707

[B156] Maeda H. Hosokawa M. Sashima T. Miyashita K. (2007b). Dietary Combination of Fucoxanthin and Fish Oil Attenuates the Weight Gain of White Adipose Tissue and Decreases Blood Glucose in Obese/diabetic KK-Ay Mice. J. Agric. Food Chem. 55 (19), 7701–7706. 10.1021/jf071569n 17715888

[B157] Maeda H. Hosokawa M. Sashima T. Murakami-Funayama K. Miyashita K. (2009). Anti-obesity and Anti-diabetic Effects of Fucoxanthin on Diet-Induced Obesity Conditions in a Murine Model. Mol. Med. Rep. 2 (6), 897–902. 10.3892/mmr_00000189 21475918

[B158] Maeda H. Kanno S. Kodate M. Hosokawa M. Miyashita K. (2015). Fucoxanthinol, Metabolite of Fucoxanthin, Improves Obesity-Induced Inflammation in Adipocyte Cells. Mar. Drugs 13 (8), 4799–4813. 10.3390/md13084799 26248075 PMC4557005

[B159] Maeda H. (2015). Nutraceutical Effects of Fucoxanthin for Obesity and Diabetes Therapy: a Review. J. Oleo Sci. 64 (2), 125–132. 10.5650/jos.ess14226 25748372

[B160] Maeda H. Tsukui T. Sashima T. Hosokawa M. Miyashita K. (2008). Seaweed Carotenoid, Fucoxanthin, as a Multi-Functional Nutrient. Asia Pac. J. Clin. Nutr. 17 Suppl 1, 196–199. 18296336

[B161] Maslowski K. M. Mackay C. R. (2011). Diet, Gut Microbiota and Immune Responses. Nat. Immunol. 12 (1), 5–9. 10.1038/ni0111-5 21169997

[B162] Matanjun P. Mohamed S. Mustapha N. M. Muhammad K. Ming C. H. (2008). Antioxidant Activities and Phenolics Content of Eight Species of Seaweeds from North Borneo. J. Appl. Phycol. 20 (4), 367–373. 10.1007/s10811-007-9264-6

[B163] Matsui M. Tanaka K. Higashiguchi N. Okawa H. Yamada Y. Tanaka K. (2016). Protective and Therapeutic Effects of Fucoxanthin against Sunburn Caused by UV Irradiation. J. Pharmacol. Sci. 132 (1), 55–64. 10.1016/j.jphs.2016.08.004 27590588

[B164] Matsumoto M. Hosokawa M. Matsukawa N. Hagio M. Shinoki A. Nishimukai M. (2010). Suppressive Effects of the Marine Carotenoids, Fucoxanthin and Fucoxanthinol on Triglyceride Absorption in Lymph Duct-Cannulated Rats. Eur. J. Nutr. 49 (4), 243–249. 10.1007/s00394-009-0078-y 19888619

[B165] Matsuno T. Ookubo M. Komori T. (1985). Carotenoids of Tunicates. III. The Structural Elucidation of Two New Marine Carotenoids, Amarouciaxanthin A and B. J. Nat. Prod. 48 (4), 606–613. 10.1021/np50040a015 3840198

[B166] Matsuzawa Y. Shimomura I. Kihara S. Funahashi T. (2003). Importance of Adipocytokines in Obesity-Related Diseases. Horm. Res. 60 Suppl 3, 56–59. 10.1159/000074502 14671398

[B167] Mei C. Zhou S. Zhu L. Ming J. Zeng F. Xu R. (2017). Antitumor Effects of Laminaria Extract Fucoxanthin on Lung Cancer. Mar. Drugs 15 (2), 39. 10.3390/md15020039 PMC5334619 28212270

[B168] Mercke Odeberg J. Lignell A. Pettersson A. Höglund P. (2003). Oral Bioavailability of the Antioxidant Astaxanthin in Humans Is Enhanced by Incorporation of Lipid Based Formulations. Eur. J. Pharm. Sci. 19 (4), 299–304. 10.1016/s0928-0987(03)00135-0 12885395

[B169] Mills C. C. Kolb E. A. Sampson V. B. (2018). Development of Chemotherapy with Cell-Cycle Inhibitors for Adult and Pediatric Cancer Therapy. Cancer Res. 78 (2), 320–325. 10.1158/0008-5472.CAN-17-2782 29311160 PMC5771851

[B170] Ming J. X. Wang Z. C. Huang Y. Ohishi H. Wu R. J. Shao Y. (2021). Fucoxanthin Extracted from Laminaria Japonica Inhibits Metastasis and Enhances the Sensitivity of Lung Cancer to Gefitinib. J. Ethnopharmacol. 265, 113302. 10.1016/j.jep.2020.113302 32860893

[B171] Mise T. Ueda M. Yasumoto T. (2011). Production of Fucoxanthin-Rich Powder from Cladosiphon Okamuranus. Adv. J. Food Sci. Technol. 3 (1), 73–76.

[B172] Mittal M. Siddiqui M. R. Tran K. Reddy S. P. Malik A. B. (2014). Reactive Oxygen Species in Inflammation and Tissue Injury. Antioxid. Redox Signal 20 (7), 1126–1167. 10.1089/ars.2012.5149 23991888 PMC3929010

[B173] Miyashita K. Beppu F. Hosokawa M. Liu X. Wang S. (2020). Bioactive Significance of Fucoxanthin and its Effective Extraction. Biocatal. Agric. Biotechnol. 26, 101639. 10.1016/j.bcab.2020.101639

[B174] Miyashita K. Hosokawa M. (2007). 12 Beneficial Health Effects of Seaweed Carotenoid. Hoboken, NJ: Fucoxanthin, 297.

[B175] Miyashita K. Hosokawa M. (2017). Fucoxanthin in the Management of Obesity and its Related Disorders. J. Funct. Foods 36, 195–202. 10.1016/j.jff.2017.07.009

[B176] Miyashita K. J. (2014). Anti-obesity Therapy by Food Component: Unique Activity of Marine Carotenoid, Fucoxanthin. Obes. Control Ther. 1 (1), 1–4. 10.15226/2374-8354/1/1/00103 25844399

[B177] Miyashita K. J. (2009). The Carotenoid Fucoxanthin from Brown Seaweed Affects Obesity. Lipid Technol. 21 (8‐9), 186–190. 10.1002/lite.200900040

[B178] Miyata M. Koyama T. Kamitani T. Toda T. Yazawa K. (2009). Anti-obesity Effect on Rodents of the Traditional Japanese Food, Tororokombu, Shaved Laminaria. Biosci. Biotechnol. Biochem. 73 (10), 2326–2328. 10.1271/bbb.90344 19809171

[B179] Miyazaki I. Asanuma M. (2008). Dopaminergic Neuron-specific Oxidative Stress Caused by Dopamine Itself. Acta Med. Okayama 62 (3), 141–150. 10.18926/AMO/30942 18596830

[B180] Miyoshi M. Ogawa A. Higurashi S. Kadooka Y. (2014). Anti-obesity Effect of Lactobacillus Gasseri SBT2055 Accompanied by Inhibition of Pro-inflammatory Gene Expression in the Visceral Adipose Tissue in Diet-Induced Obese Mice. Eur. J. Nutr. 53 (2), 599–606. 10.1007/s00394-013-0568-9 23917447

[B181] Mohamadnia S. Tavakoli O. Faramarzi M. A. (2021). Enhancing Production of Fucoxanthin by the Optimization of Culture Media of the Microalga Tisochrysis Lutea. Aquaculture 533, 736074. 10.1016/j.aquaculture.2020.736074

[B182] Mohamadnia S. Tavakoli O. Faramarzi M. A. Shamsollahi Z. (2020). Production of Fucoxanthin by the Microalga Tisochrysis Lutea: a Review of Recent Developments. Aquaculture 516, 734637. 10.1016/j.aquaculture.2019.734637

[B183] Mohammed M. T. Kadhim S. M. Jassimand A. N. Abbas S. J. (2015). Free Radicals and Human Health. Int. J. Innovation Sci. Res. 4 (6), 218–223. 10.4103/2278-0513.148915

[B184] Mohibbullah M. Haque M. N. Khan M. N. A. Park I.-S. Moon I. S. Hong Y.-K. (2018). Neuroprotective Effects of Fucoxanthin and its Derivative Fucoxanthinol from the Phaeophyte Undaria Pinnatifida Attenuate Oxidative Stress in Hippocampal Neurons. J. Appl. Phycol. 30 (6), 3243–3252. 10.1007/s10811-018-1458-6

[B185] Møller I. M. Jensen P. E. Hansson A. (2007). Oxidative Modifications to Cellular Components in Plants. Annu. Rev. Plant Biol. 58, 459–481. 10.1146/annurev.arplant.58.032806.103946 17288534

[B186] Mordecai E. A. Paaijmans K. P. Johnson L. R. Balzer C. Ben-Horin T. De Moor E. (2013). Optimal Temperature for Malaria Transmission Is Dramatically Lower Than Previously Predicted. Ecol. Lett. 16 (1), 22–30. 10.1111/ele.12015 23050931

[B187] Moreau D. Tomasoni C. Jacquot C. Kaas R. Le Guedes R. Cadoret J. P. (2006). Cultivated Microalgae and the Carotenoid Fucoxanthin from Odontella Aurita as Potent Anti-proliferative Agents in Bronchopulmonary and Epithelial Cell Lines. Environ. Toxicol. Pharmacol. 22 (1), 97–103. 10.1016/j.etap.2006.01.004 21783694

[B188] Mori K. Ooi T. Hiraoka M. Oka N. Hamada H. Tamura M. (2004). Fucoxanthin and its Metabolites in Edible Brown Algae Cultivated in Deep Seawater. Mar. Drugs 2 (2), 63–72. 10.3390/md202063

[B189] Mousavi Nadushan R. Hosseinzade I. (2020). Optimization of Production and Antioxidant Activity of Fucoxanthin from Marine Haptophyte Algae. Isochrysis galbana 19 (6), 2901–2908.

[B190] Muradian Kh. Vaiserman A. Min K. J. Fraifeld V. E. (2015). Fucoxanthin and Lipid Metabolism: A Minireview. Nutr. Metab. Cardiovasc Dis. 25 (10), 891–897. 10.1016/j.numecd.2015.05.010 26141943

[B191] Murakami C. Takemura M. Sugiyama Y. Kamisuki S. Asahara H. Kawasaki M. (2002). Vitamin A-Related Compounds, All-Trans Retinal and Retinoic Acids, Selectively Inhibit Activities of Mammalian Replicative DNA Polymerases. Biochim. Biophys. Acta 1574 (1), 85–92. 10.1016/s0167-4781(01)00348-7 11955616

[B192] Nabeshima K. Inoue T. Shimao Y. Sameshima T. (2002). Matrix Metalloproteinases in Tumor Invasion: Role for Cell Migration. Pathol. Int. 52 (4), 255–264. 10.1046/j.1440-1827.2002.01343.x 12031080

[B193] Nakazawa Y. Sashima T. Hosokawa M. Miyashita K. (2009). Comparative Evaluation of Growth Inhibitory Effect of Stereoisomers of Fucoxanthin in Human Cancer Cell Lines. J. Funct. Foods 1 (1), 88–97. 10.1016/j.jff.2008.09.015

[B194] Namkoong S. Joo H.-M. Jang S.-A. Kim Y.-J. Kim T.-S. Sohn E.-H. (2012). Suppressive Effects of Fucoxanthin on Degranulation in IgE-Antigen Complex-Stimulated RBL-2H3 Cells. Korean J. Plant Resour. 25 (3), 339–345. 10.7732/kjpr.2012.25.3.339

[B195] Narang S. Gibson D. Wasan A. D. Ross E. L. Michna E. Nedeljkovic S. S. (2008). Efficacy of Dronabinol as an Adjuvant Treatment for Chronic Pain Patients on Opioid Therapy. J. Pain 9 (3), 254–264. 10.1016/j.jpain.2007.10.018 18088560

[B196] Ngo D.-H. Wijesekara I. Vo T.-S. Van Ta Q. Kim S.-K. (2011). Marine Food-Derived Functional Ingredients as Potential Antioxidants in the Food Industry: An Overview. Food Res. Int. 44 (2), 523–529. 10.1016/j.foodres.2010.12.030

[B197] Nicot C. (2005). Current Views in HTLV-I-Associated Adult T-Cell Leukemia/lymphoma. Am. J. Hematol. 78 (3), 232–239. 10.1002/ajh.20307 15726602

[B198] Nie J. Chen D. Lu Y. Dai Z. (2021). Effects of Various Blanching Methods on Fucoxanthin Degradation Kinetics, Antioxidant Activity, Pigment Composition, and Sensory Quality of Sargassum Fusiforme. Lwt 143, 111179. 10.1016/j.lwt.2021.111179

[B199] Nieto-Vazquez I. Fernández-Veledo S. Krämer D. K. Vila-Bedmar R. Garcia-Guerra L. Lorenzo M. (2008). Insulin Resistance Associated to Obesity: the Link TNF-Alpha. Arch. Physiol. Biochem. 114 (3), 183–194. 10.1080/13813450802181047 18629684

[B200] Nigro E. Scudiero O. Monaco M. L. Palmieri A. Mazzarella G. Costagliola C. (2014). New Insight into Adiponectin Role in Obesity and Obesity-Related Diseases. 10.1155/2014/658913 PMC4109424 25110685

[B201] Nishino H. (1998). Cancer Prevention by Carotenoids. Mutat. Res. 402 (1-2), 159–163. 10.1016/s0027-5107(97)00293-5 9675267

[B202] Nomura T. Kikuchi M. Kubodera A. Kawakami Y. (1997). Proton-donative Antioxidant Activity of Fucoxanthin with 1,1-Diphenyl-2-Picrylhydrazyl (DPPH). Biochem. Mol. Biol. Int. 42 (2), 361–370. 10.1080/15216549700202761 9238535

[B203] Noor A. M. Kinyoki D. K. Mundia C. W. Kabaria C. W. Mutua J. W. Alegana V. A. (2014). The Changing Risk of Plasmodium Falciparum Malaria Infection in Africa: 2000-10: a Spatial and Temporal Analysis of Transmission Intensity. Lancet 383 (9930), 1739–1747. 10.1016/s0140-6736(13)62566-0 24559537 PMC4030588

[B204] Okada T. Mizuno Y. Sibayama S. Hosokawa M. Miyashita K. (2011). Antiobesity Effects of Undaria Lipid Capsules Prepared with Scallop Phospholipids. J. Food Sci. 76 (1), H2–H6. 10.1111/j.1750-3841.2010.01878.x 21535684

[B205] Okuzumi J. Nishino H. Murakoshi M. Iwashima A. Tanaka Y. Yamane T. (1990). Inhibitory Effects of Fucoxanthin, a Natural Carotenoid, on N-Myc Expression and Cell Cycle Progression in Human Malignant Tumor Cells. Cancer Lett. 55 (1), 75–81. 10.1016/0304-3835(90)90068-9 2245414

[B206] Okuzumi J. Takahashi T. Yamane T. Kitao Y. Inagake M. Ohya K. (1993). Inhibitory Effects of Fucoxanthin, a Natural Carotenoid, on N-Ethyl-N'-Nitro-N-Nitrosoguanidine-Induced Mouse Duodenal Carcinogenesis. Cancer Lett. 68 (2-3), 159–168. 10.1016/0304-3835(93)90142-v 8443788

[B207] Oshima S. Inakuma T. Narisawa T. (1999). Absorption and Distribution of Lycopene in Rat Colon. J. Nutr. Sci. Vitaminol. (Tokyo) 45 (1), 129–134. 10.3177/jnsv.45.129 10360247

[B208] Pangestuti R. Kim S. K. (2010). Neuroprotective Properties of Chitosan and its Derivatives. Mar. Drugs 8 (7), 2117–2128. 10.3390/md8072117 20714426 PMC2920545

[B209] Pangestuti R. Vo T. S. Ngo D. H. Kim S. K. (2013). Fucoxanthin Ameliorates Inflammation and Oxidative Reponses in Microglia. J. Agric. Food Chem. 61 (16), 3876–3883. 10.1021/jf400015k 23551304

[B210] Park H. J. Lee M. K. Park Y. B. Shin Y. C. Choi M. S. (2011). Beneficial Effects of Undaria Pinnatifida Ethanol Extract on Diet-Induced-Insulin Resistance in C57BL/6J Mice. Food Chem. Toxicol. 49 (4), 727–733. 10.1016/j.fct.2010.11.032 21146577

[B211] Park Y. K. Ledesma-Amaro R. Nicaud J. M. (2019). De Novo Biosynthesis of Odd-Chain Fatty Acids in Yarrowia Lipolytica Enabled by Modular Pathway Engineering. Front. Bioeng. Biotechnol. 7, 484. 10.3389/fbioe.2019.00484 32039184 PMC6987463

[B212] Peng J. Yuan J. P. Wu C. F. Wang J. H. (2011). Fucoxanthin, a Marine Carotenoid Present in Brown Seaweeds and Diatoms: Metabolism and Bioactivities Relevant to Human Health. Mar. Drugs 9 (10), 1806–1828. 10.3390/md9101806 22072997 PMC3210606

[B213] Peraman M. Nachimuthu S. (2019). Identification and Quantification of Fucoxanthin in Selected Carotenoid-Producing Marine Microalgae and Evaluation for Their Chemotherapeutic Potential. Phcog Mag. 15 (64), 243. 10.4103/pm.pm_64_19

[B214] Pérez M. Falqué E. Domínguez H. (2016). Antimicrobial Action of Compounds from Marine Seaweed. Mar. Drugs 14 (3), 52. 10.3390/md14030052 PMC4820306 27005637

[B215] Petri D. Lundebye A. K. (2007). Tissue Distribution of Astaxanthin in Rats Following Exposure to Graded Levels in the Feed. Comp. Biochem. Physiol. C Toxicol. Pharmacol. 145 (2), 202–209. 10.1016/j.cbpc.2006.12.008 17257901

[B216] Pinto E. Sigaud-kutner T. C. S. Leitao M. A. S. Okamoto O. K. Morse D. Colepicolo P. (2003). Heavy Metal-Induced Oxidative Stress in Algae1. J. Phycol. 39 (6), 1008–1018. 10.1111/j.0022-3646.2003.02-193.x

[B217] Poor C. L. Bierer T. L. Merchen N. R. Fahey G. C. Jr Erdman J. W. Jr (1993). The Accumulation of Alpha- and Beta-Carotene in Serum and Tissues of Preruminant Calves Fed Raw and Steamed Carrot Slurries. J. Nutr. 123 (7), 1296–1304. 10.1093/jn/123.7.1296 8320568

[B218] Qin J. Li Y. Cai Z. Li S. Zhu J. Zhang F. (2012). A Metagenome-wide Association Study of Gut Microbiota in Type 2 Diabetes. Nature 490 (7418), 55–60. 10.1038/nature11450 23023125

[B219] Quigley E. M. (2013). Gut Bacteria in Health and Disease. Gastroenterol. Hepatol. 9 (9), 560–569. PMC3983973 24729765

[B220] Raisz L. G. Rodan G. A. (2003). Pathogenesis of Osteoporosis. Endocrinol. Metab. Clin. North Am. 32 (1), 15–24. 10.1016/s0889-8529(02)00055-5 12699290

[B221] Rajauria G. Abu-Ghannam N. (2013). Isolation and Partial Characterization of Bioactive Fucoxanthin from Himanthalia Elongata Brown Seaweed: A TLC-Based Approach. Int. J. Anal. Chem. 2013, 802573. 10.1155/2013/802573 23762062 PMC3665216

[B222] Rajauria G. Foley B. Abu-Ghannam N. (2017). Characterization of Dietary Fucoxanthin from Himanthalia Elongata Brown Seaweed. Food Res. Int. 99 (Pt 3), 995–1001. 10.1016/j.foodres.2016.09.023 28865626

[B223] Ramalingam R. Vaiyapuri M. (2013). Effects of Umbelliferone on Lipid Peroxidation and Antioxidant Status in Diethylnitrosamine-Induced Hepatocellular Carcinoma. J. Acute Med. 3 (3), 73–82. 10.1016/j.jacme.2013.05.001

[B224] Rao J. S. (2003). Molecular Mechanisms of Glioma Invasiveness: the Role of Proteases. Nat. Rev. Cancer 3 (7), 489–501. 10.1038/nrc1121 12835669

[B225] Raposo M. F. De Morais A. M. De Morais R. M. (2015). Carotenoids from Marine Microalgae: A Valuable Natural Source for the Prevention of Chronic Diseases. Mar. Drugs 13 (8), 5128–5155. 10.3390/md13085128 26287216 PMC4557017

[B226] Reddy J. K. Rao M. S. (2006). Lipid Metabolism and Liver Inflammation. II. Fatty Liver Disease and Fatty Acid Oxidation. Am. J. Physiol. Gastrointest. Liver Physiol. 290 (5), G852–G858. 10.1152/ajpgi.00521.2005 16603729

[B227] Remely M. Hippe B. Zanner J. Aumueller E. Brath H. Haslberger A. G. (2016). Gut Microbiota of Obese, Type 2 Diabetic Individuals Is Enriched in Faecalibacterium Prausnitzii, Akkermansia Muciniphila and Peptostreptococcus Anaerobius after Weight Loss. Endocr. Metab. Immune Disord. Drug Targets 16 (2), 99–106. 10.2174/1871530316666160831093813 27577947

[B228] Ren L. Sun D. Zhou X. Yang Y. Huang X. Li Y. (2019). Chronic Treatment with the Modified Longdan Xiegan Tang Attenuates Olanzapine-Induced Fatty Liver in Rats by Regulating Hepatic De Novo Lipogenesis and Fatty Acid Beta-Oxidation-Associated Gene Expression Mediated by SREBP-1c, PPAR-Alpha and AMPK-Alpha. J. Ethnopharmacol. 232, 176–187. 10.1016/j.jep.2018.12.034 30590197

[B229] Rhee C. W. Lee J. Oh S. Choi N. K. Park B. J. (2012). Use of Bisphosphonate and Risk of Atrial Fibrillation in Older Women with Osteoporosis. Osteoporos. Int. 23 (1), 247–254. 10.1007/s00198-011-1608-z 21431993

[B230] Rijstenbil J. (2003). Effects of UVB Radiation and Salt Stress on Growth, Pigments and Antioxidative Defence of the Marine Diatom Cylindrotheca Closterium. Mar. Ecol. Prog. Ser. 254, 37–48. 10.3354/meps254037

[B231] Roehlecke C. Schaller A. Knels L. Funk R. H. (2009). The Influence of Sublethal Blue Light Exposure on Human RPE Cells. Mol. Vis. 15, 1929–1938. 19784391 PMC2751800

[B232] Rokkaku T. Kimura R. Ishikawa C. Yasumoto T. Senba M. Kanaya F. (2013). Anticancer Effects of Marine Carotenoids, Fucoxanthin and its Deacetylated Product, Fucoxanthinol, on Osteosarcoma. Int. J. Oncol. 43 (4), 1176–1186. 10.3892/ijo.2013.2019 23857515

[B233] Ross J. (2002). The Biology of the Macrophage. Burlington, MA: ScienceOpen, Inc.

[B234] Rowland I. Gibson G. Heinken A. Scott K. Swann J. Thiele I. (2018). Gut Microbiota Functions: Metabolism of Nutrients and Other Food Components. Eur. J. Nutr. 57 (1), 1–24. 10.1007/s00394-017-1445-8 PMC5847071 28393285

[B235] Sachindra N. M. Airanthi M. K. Hosokawa M. Miyashita K. (2010). Radical Scavenging and Singlet Oxygen Quenching Activity of Extracts from Indian Seaweeds. J. Food Sci. Technol. 47 (1), 94–99. 10.1007/s13197-010-0022-4 23572608 PMC3550984

[B236] Sachindra N. M. Sato E. Maeda H. Hosokawa M. Niwano Y. Kohno M. (2007). Radical Scavenging and Singlet Oxygen Quenching Activity of Marine Carotenoid Fucoxanthin and its Metabolites. J. Agric. Food Chem. 55 (21), 8516–8522. 10.1021/jf071848a 17894451

[B237] Sailaja Rao P. Kalva S. Yerramilli A. Mamidi S. (2011). Free Radicals and Tissue Damage: Role of Antioxidants. Free Radicals Antioxidants 1 (4), 2–7. 10.5530/ax.2011.4.2

[B238] Sakai S. Sugawara T. Hirata T. (2011). Inhibitory Effect of Dietary Carotenoids on Dinitrofluorobenzene-Induced Contact Hypersensitivity in Mice. Biosci. Biotechnol. Biochem. 75 (5), 1013–1015. 10.1271/bbb.110104 21597166

[B239] Sakai S. Sugawara T. Matsubara K. Hirata T. (2009). Inhibitory Effect of Carotenoids on the Degranulation of Mast Cells via Suppression of Antigen-Induced Aggregation of High Affinity IgE Receptors. J. Biol. Chem. 284 (41), 28172–28179. 10.1074/jbc.M109.001099 19700409 PMC2788868

[B240] Sangeetha R. K. Bhaskar N. Baskaran V. (2009). Comparative Effects of Beta-Carotene and Fucoxanthin on Retinol Deficiency Induced Oxidative Stress in Rats. Mol. Cell. Biochem. 331 (1-2), 59–67. 10.1007/s11010-009-0145-y 19421712

[B241] Sangeetha R. K. Bhaskar N. Divakar S. Baskaran V. (2010). Bioavailability and Metabolism of Fucoxanthin in Rats: Structural Characterization of Metabolites by LC-MS (APCI). Mol. Cell. Biochem. 333 (1-2), 299–310. 10.1007/s11010-009-0231-1 19701609

[B242] Savira A. D. R. Amin M. N. G. Alamsjah M. A. (2021). The Effect of Different Type of Solvents on the Antioxidant Activity of Fucoxanthin Extract from Brown Seaweed Sargassum Duplicatum. IOP Conf. Ser. Earth Environ. Sci. 718, 012010. 10.1088/1755-1315/718/1/012010

[B243] Schilter B. Scholz G. Seefelder W. (2010). Fatty Acid Esters of Chloropropanols and Related Compounds in Food: Toxicological Aspects. Eur. J. Lipid Sci. Technol. 113 (3), 309–313. 10.1002/ejlt.201000311

[B244] Schneider C. Teufel A. Yevsa T. Staib F. Hohmeyer A. Walenda G. (2012). Adaptive Immunity Suppresses Formation and Progression of Diethylnitrosamine-Induced Liver Cancer. Gut 61 (12), 1733–1743. 10.1136/gutjnl-2011-301116 22267597 PMC4533880

[B245] Schreurs M. Kuipers F. Van Der Leij F. R. (2010). Regulatory Enzymes of Mitochondrial Beta-Oxidation as Targets for Treatment of the Metabolic Syndrome. Obes. Rev. 11 (5), 380–388. 10.1111/j.1467-789X.2009.00642.x 19694967

[B246] Schweigert F. J. Buchholz I. Schuhmacher A. Gropp J. (2001). Effect of Dietary Beta-Carotene on the Accumulation of Beta-Carotene and Vitamin A in Plasma and Tissues of Gilts. Reprod. Nutr. Dev. 41 (1), 47–55. 10.1051/rnd:2001111 11368244

[B247] Scott K. P. Antoine J. M. Midtvedt T. Van Hemert S. (2015). Manipulating the Gut Microbiota to Maintain Health and Treat Disease. Microb. Ecol. Health Dis. 26 (1), 25877. 10.3402/mehd.v26.25877 25651995 PMC4315778

[B248] Scott K. P. Gratz S. W. Sheridan P. O. Flint H. J. Duncan S. H. (2013). The Influence of Diet on the Gut Microbiota. Pharmacol. Res. 69 (1), 52–60. 10.1016/j.phrs.2012.10.020 23147033

[B249] Seon-Jin L. J. Se-Kyung B. Kwang-Soon L. Seung N. Hee-Jun N. Kwon-Soo H. (2003). Astaxanthin Inhibits Nitric Oxide Production and Inflammatory Gene Expression by Suppressing IkB Kinase-dependent NF-kB Activation. Mol. Cells. 16, 97–105. 14503852

[B250] Shang Q. Song G. Zhang M. Shi J. Xu C. Hao J. (2017). Dietary Fucoidan Improves Metabolic Syndrome in Association with Increased Akkermansia Population in the Gut Microbiota of High-Fat Diet-Fed Mice. J. Funct. Foods 28, 138–146. 10.1016/j.jff.2016.11.002

[B251] Shannon E. Abu-Ghannam N. (2016). Antibacterial Derivatives of Marine Algae: An Overview of Pharmacological Mechanisms and Applications. Mar. Drugs 14 (4), 81. 10.3390/md14040081 PMC4849085 27110798

[B252] Shannon E. Abu-Ghannam N. (2017). Optimisation of Fucoxanthin Extraction from Irish Seaweeds by Response Surface Methodology. J. Appl. Phycol. 29 (2), 1027–1036. 10.1007/s10811-016-0983-4

[B253] Shimoda H. Tanaka J. Shan S. J. Maoka T. (2010). Anti-pigmentary Activity of Fucoxanthin and its Influence on Skin mRNA Expression of Melanogenic Molecules. J. Pharm. Pharmacol. 62 (9), 1137–1145. 10.1111/j.2042-7158.2010.01139.x 20796192

[B254] Shiratori K. Ohgami K. Ilieva I. Jin X. H. Koyama Y. Miyashita K. (2005). Effects of Fucoxanthin on Lipopolysaccharide-Induced Inflammation In Vitro and In Vivo. Exp. Eye Res. 81 (4), 422–428. 10.1016/j.exer.2005.03.002 15950219

[B255] Shirouchi B. Nagao K. Umegatani M. Shiraishi A. Morita Y. Kai S. (2016). Probiotic Lactobacillus Gasseri SBT2055 Improves Glucose Tolerance and Reduces Body Weight Gain in Rats by Stimulating Energy Expenditure. Br. J. Nutr. 116 (3), 451–458. 10.1017/S0007114516002245 27267802

[B256] Showalter L. A. Weinman S. A. Østerlie M. Lockwood S. F. (2004). Plasma Appearance and Tissue Accumulation of Non-esterified, Free Astaxanthin in C57BL/6 Mice after Oral Dosing of a Disodium Disuccinate Diester of Astaxanthin (Heptax). Comp. Biochem. Physiol. C Toxicol. Pharmacol. 137 (3), 227–236. 10.1016/j.cca.2003.12.006 15171947

[B257] Sidhu A. B. Uhlemann A. C. Valderramos S. G. Valderramos J. C. Krishna S. Fidock D. A. (2006). Decreasing Pfmdr1 Copy Number in Plasmodium Falciparum Malaria Heightens Susceptibility to Mefloquine, Lumefantrine, Halofantrine, Quinine, and Artemisinin. J. Infect. Dis. 194 (4), 528–535. 10.1086/507115 16845638 PMC2978021

[B258] Sirtori C. R. Galli C. Anderson J. W. Arnoldi A. (2009). Nutritional and Nutraceutical Approaches to Dyslipidemia and Atherosclerosis Prevention: Focus on Dietary Proteins. Atherosclerosis 203 (1), 8–17. 10.1016/j.atherosclerosis.2008.06.019 18687434

[B259] Sivagnanam S. P. Yin S. Choi J. H. Park Y. B. Woo H. C. Chun B. S. (2015). Biological Properties of Fucoxanthin in Oil Recovered from Two Brown Seaweeds Using Supercritical CO2 Extraction. Mar. Drugs 13 (6), 3422–3442. 10.3390/md13063422 26035021 PMC4483637

[B260] Snow R. W. Guerra C. A. Noor A. M. Myint H. Y. Hay S. I. (2005). The Global Distribution of Clinical Episodes of Plasmodium Falciparum Malaria. Nature 434 (7030), 214–217. 10.1038/nature03342 15759000 PMC3128492

[B261] Soares Nda. C. Teodoro A. J. Lotsch P. F. Granjeiro J. M. Borojevic R. (2015). Anticancer Properties of Carotenoids in Prostate Cancer. A Review. Histol. Histopathol. 30 (10), 1143–1154. 10.14670/HH-11-635 26058846

[B262] Sporn M. B. Suh N. (2000). Chemoprevention of Cancer. Carcinogenesis 21 (3), 525–530. 10.1093/carcin/21.3.525 10688873

[B263] Stengel D. B. Dring M. J. (1998). Seasonal Variation in the Pigment Content and Photosynthesis of Different Thallus Regions of Ascophyllum Nodosum (Fucales, Phaeophyta) in Relation to Position in the Canopy. Phycologia 37 (4), 259–268. 10.2216/i0031-8884-37-4-259.1

[B264] Strand A. Herstad O. Liaaen-Jensen S. (1998). Fucoxanthin Metabolites in Egg Yolks of Laying Hens. Comp. Biochem. Physiol. A Mol. Integr. Physiol. 119 (4), 963–974. 10.1016/s1095-6433(98)00011-7 9773489

[B265] Streckaite S. Gardian Z. Li F. Pascal A. A. Litvin R. Robert B. (2018). Pigment Configuration in the Light-Harvesting Protein of the Xanthophyte Alga Xanthonema Debile. Photosynth Res. 138 (2), 139–148. 10.1007/s11120-018-0557-1 30006883

[B266] Sugawara T. Baskaran V. Tsuzuki W. Nagao A. (2002). Brown Algae Fucoxanthin Is Hydrolyzed to Fucoxanthinol during Absorption by Caco-2 Human Intestinal Cells and Mice. J. Nutr. 132 (5), 946–951. 10.1093/jn/132.5.946 11983819

[B267] Sugawara T. Matsubara K. Akagi R. Mori M. Hirata T. (2006). Antiangiogenic Activity of Brown Algae Fucoxanthin and its Deacetylated Product, Fucoxanthinol. J. Agric. Food Chem. 54 (26), 9805–9810. 10.1021/jf062204q 17177505

[B268] Sugiura Y. Kinoshita Y. Usui M. Tanaka R. Matsushita T. Miyata M. (2016). The Suppressive Effect of a Marine Carotenoid, Fucoxanthin, on Mouse Ear Swelling through Regulation of Activities and mRNA Expression of Inflammation-Associated Enzymes. Fstr 22 (2), 227–234. 10.3136/fstr.22.227

[B269] Sun X. Xu Y. Zhao L. Yan H. Wang S. Wang D. (2018). The Stability and Bioaccessibility of Fucoxanthin in Spray-Dried Microcapsules Based on Various Biopolymers. RSC Adv. 8 (61), 35139–35149. 10.1039/c8ra05621h 35547077 PMC9087948

[B270] Sun X. Zhao H. Liu Z. Sun X. Zhang D. Wang S. (2020). Modulation of Gut Microbiota by Fucoxanthin during Alleviation of Obesity in High-Fat Diet-Fed Mice. J. Agric. Food Chem. 68 (18), 5118–5128. 10.1021/acs.jafc.0c01467 32309947

[B271] Susanto E. Fahmi A. S. Agustini T. W. Rosyadi S. Wardani A. D. (2017). Effects of Different Heat Processing on Fucoxanthin, Antioxidant Activity and Colour of Indonesian Brown Seaweeds. IOP Conf. Ser. Earth Environ. Sci. 55, 012063. 10.1088/1755-1315/55/1/012063

[B272] Tabatabaei-Malazy O. Salari P. Khashayar P. Larijani B. (2017). New Horizons in Treatment of Osteoporosis. Daru 25 (1), 2. 10.1186/s40199-017-0167-z 28173850 PMC5297185

[B273] Takaichi S. (2011). Carotenoids in Algae: Distributions, Biosyntheses and Functions. Mar. Drugs 9 (6), 1101–1118. 10.3390/md9061101 21747749 PMC3131562

[B274] Tamama K. J. (2020). Potential Benefits of Dietary Seaweeds as Protection against COVID-19. Nutr. Rev. 79 (7), 814–823. 10.1093/nutrit/nuaa126 PMC7798825 33341894

[B275] Terasaki M. Iida T. Kikuchi F. Tamura K. Endo T. Kuramitsu Y. (2019). Fucoxanthin Potentiates Anoikis in Colon Mucosa and Prevents Carcinogenesis in AOM/DSS Model Mice. J. Nutr. Biochem. 64, 198–205. 10.1016/j.jnutbio.2018.10.007 30530259

[B276] Terasaki M. Uehara O. Ogasa S. Sano T. Kubota A. Kojima H. (2021). Alteration of Fecal Microbiota by Fucoxanthin Results in Prevention of Colorectal Cancer in AOM/DSS Mice. Carcinogenesis 42 (2), 210–219. 10.1093/carcin/bgaa100 32940665

[B277] Terasaki M. Ikuta M. Kojima H. Tanaka T. Maeda H. Miyashita K. (2020). Dietary Fucoxanthin Induces Anoikis in Colorectal Adenocarcinoma by Suppressing Integrin Signaling in a Murine Colorectal Cancer Model. J. Clin. Med. 9 (1), 90. 10.3390/jcm9010090 PMC7019251 31905803

[B278] Tetko I. V. Gasteiger J. Todeschini R. Mauri A. Livingstone D. Ertl P. (2005). Virtual Computational Chemistry Laboratory-Ddesign and Description. J. Comput. Aided Mol. Des. 19 (6), 453–463. 10.1007/s10822-005-8694-y 16231203

[B279] Tirone T. A. Brunicardi F. C. (2001). Overview of Glucose Regulation. World J. Surg. 25 (4), 461–467. 10.1007/s002680020338 11344399

[B280] Torres M. D. Flórez-Fernández N. Simón-Vázquez R. Giménez-Abián J. F. Díaz J. F. González-Fernández Á. (2020). Fucoidans: The Importance of Processing on Their Anti-tumoral Properties. Algal Res. 45, 101748. 10.1016/j.algal.2019.101748

[B281] Tsukui T. Konno K. Hosokawa M. Maeda H. Sashima T. Miyashita K. (2007). Fucoxanthin and Fucoxanthinol Enhance the Amount of Docosahexaenoic Acid in the Liver of KKAy Obese/diabetic Mice. J. Agric. Food Chem. 55 (13), 5025–5029. 10.1021/jf070110q 17536824

[B282] Tsukui T. Baba N. Hosokawa M. Sashima T. Miyashita K. (2009). Enhancement of Hepatic Docosahexaenoic Acid and Arachidonic Acid Contents in C57BL/6J Mice by Dietary Fucoxanthin. Fish. Sci. 75 (1), 261–263. 10.1007/s12562-008-0018-4

[B283] Tsushima M. Maoka T. Katsuyama M. Kozuka M. Matsuno T. Tokuda H. (1995). Inhibitory Effect of Natural Carotenoids on Epstein-Barr Virus Activation Activity of a Tumor Promoter in Raji Cells. A Screening Study for Anti-tumor Promoters. Biol. Pharm. Bull. 18 (2), 227–233. 10.1248/bpb.18.227 7742789

[B284] Tsushima M. (2007). “Chapter 8 Carotenoids in Sea Urchins,” in Edible Sea Urchins: Biology and Ecology Lawrence (Amsterdam, Netherlands: Elsevier). 10.1016/s0167-9309(07)80072-x

[B285] Tucci P. Bagetta G. (2008). How to Study Neuroprotection? Cell. Death Differ. 15 (6), 1084–1085. 10.1038/cdd.2008.32

[B286] Turnbaugh P. J. Bäckhed F. Fulton L. Gordon J. I. (2008). Diet-induced Obesity Is Linked to Marked but Reversible Alterations in the Mouse Distal Gut Microbiome. Cell. Host Microbe 3 (4), 213–223. 10.1016/j.chom.2008.02.015 18407065 PMC3687783

[B287] Turnbaugh P. J. Ley R. E. Mahowald M. A. Magrini V. Mardis E. R. Gordon J. I. (2006). An Obesity-Associated Gut Microbiome with Increased Capacity for Energy Harvest. Nature 444 (7122), 1027–1031. 10.1038/nature05414 17183312

[B288] Turrens J. F. (2003). Mitochondrial Formation of Reactive Oxygen Species. J. Physiol. 552 (Pt 2), 335–344. 10.1113/jphysiol.2003.049478 14561818 PMC2343396

[B289] Tyagi R. K. Gleeson P. J. Arnold L. Tahar R. Prieur E. Decosterd L. (2018). High-level Artemisinin-Resistance with Quinine Co-resistance Emerges in P. Falciparum Malaria under In Vivo Artesunate Pressure. BMC Med. 16 (1), 181. 10.1186/s12916-018-1156-x 30269689 PMC6166299

[B290] Urikura I. Sugawara T. Hirata T. (2011). Protective Effect of Fucoxanthin against UVB-Induced Skin Photoaging in Hairless Mice. Biosci. Biotechnol. Biochem. 75 (4), 757–760. 10.1271/bbb.110040 21512228

[B291] Velmurugan G. Ramprasath T. Gilles M. Swaminathan K. Ramasamy S. (2017). Gut Microbiota, Endocrine-Disrupting Chemicals, and the Diabetes Epidemic. Trends Endocrinol. Metab. 28 (8), 612–625. 10.1016/j.tem.2017.05.001 28571659

[B292] Viera I. Pérez-Gálvez A. Roca M. (2018). Bioaccessibility of Marine Carotenoids. Mar. Drugs 16 (10), 397. 10.3390/md16100397 PMC6213429 30360450

[B293] Vijay K. Sowmya P. R. Arathi B. P. Shilpa S. Shwetha H. J. Raju M. (2018). Low-dose Doxorubicin with Carotenoids Selectively Alters Redox Status and Upregulates Oxidative Stress-Mediated Apoptosis in Breast Cancer Cells. Food Chem. Toxicol. 118, 675–690. 10.1016/j.fct.2018.06.027 29920287

[B294] Vluggens A. Andreoletti P. Viswakarma N. Jia Y. Matsumoto K. Kulik W. (2010). Reversal of Mouse Acyl-CoA Oxidase 1 (ACOX1) Null Phenotype by Human ACOX1b Isoform [corrected]. Lab. Invest. 90 (5), 696–708. 10.1038/labinvest.2010.46 20195242

[B295] Vrieze A. Van Nood E. Holleman F. Salojärvi J. Kootte R. S. Bartelsman J. F. (2012). Transfer of Intestinal Microbiota from Lean Donors Increases Insulin Sensitivity in Individuals with Metabolic Syndrome. Gastroenterology 143 (4), 913–e7. 10.1053/j.gastro.2012.06.031 22728514

[B296] Wakefield D. Chang J. H. (2005). Epidemiology of Uveitis. Int. Ophthalmol. Clin. 45 (2), 1–13. 10.1097/01.iio.0000155938.83083.94 15791154

[B297] Walker C. G. Zariwala M. G. Holness M. J. Sugden M. C. (2007). Diet, Obesity and Diabetes: a Current Update. Clin. Sci. (Lond) 112 (2), 93–111. 10.1042/CS20060150 17155931

[B298] Walsh N. C. Crotti T. N. Goldring S. R. Gravallese E. M. (2005). Rheumatic Diseases: the Effects of Inflammation on Bone. Immunol. Rev. 208 (1), 228–251. 10.1111/j.0105-2896.2005.00338.x 16313352

[B299] Wang J. Ma Y. Yang J. Jin L. Gao Z. Xue L. (2019). Fucoxanthin Inhibits Tumour-Related Lymphangiogenesis and Growth of Breast Cancer. J. Cell. Mol. Med. 23 (3), 2219–2229. 10.1111/jcmm.14151 30648805 PMC6378177

[B300] Wang L. Zeng Y. Liu Y. Hu X. Li S. Wang Y. (2014). Fucoxanthin Induces Growth Arrest and Apoptosis in Human Bladder Cancer T24 Cells by Up-Regulation of P21 and Down-Regulation of Mortalin. Acta Biochim. Biophys. Sin. (Shanghai) 46 (10), 877–884. 10.1093/abbs/gmu080 25187415

[B301] Wang L. J. Fan Y. Parsons R. L. Hu G. R. Zhang P. Y. Li F. L. (2018). A Rapid Method for the Determination of Fucoxanthin in Diatom. Mar. Drugs 16 (1), 33. 10.3390/md16010033 PMC5793081 29361768

[B302] Wang J. Chen S. Xu S. Yu X. Ma D. Hu X. (2012). In Vivo induction of Apoptosis by Fucoxanthin, a Marine Carotenoid, Associated with Down-Regulating STAT3/EGFR Signaling in Sarcoma 180 (S180) Xenografts-Bearing Mice. Mar. Drugs 10 (9), 2055–2068. 10.3390/md10092055 23118721 PMC3475273

[B303] Wang T. Cai G. Qiu Y. Fei N. Zhang M. Pang X. (2012). Structural Segregation of Gut Microbiota between Colorectal Cancer Patients and Healthy Volunteers. ISME J. 6 (2), 320–329. 10.1038/ismej.2011.109 21850056 PMC3260502

[B304] Wang X. Chen K. Wang S. Wang Q. Hu Y. Yin F. (2022). Distribution of Tyrosol Fatty Acid Esters in the Gastrointestinal Tracts of Mice and Their Hydrolysis Characteristics by Gut Microbiota. Food Funct. 13 (5), 2998–3008. 10.1039/d1fo04029d 35195115

[B305] Wenzel A. Grimm C. Samardzija M. Remé C. E. (2005). Molecular Mechanisms of Light-Induced Photoreceptor Apoptosis and Neuroprotection for Retinal Degeneration. Prog. Retin Eye Res. 24 (2), 275–306. 10.1016/j.preteyeres.2004.08.002 15610977

[B306] Wingerath T. Stahl W. Sies H. (1995). Beta-Cryptoxanthin Selectively Increases in Human Chylomicrons upon Ingestion of Tangerine Concentrate Rich in Beta-Cryptoxanthin Esters. Arch. Biochem. Biophys. 324 (2), 385–390. 10.1006/abbi.1995.0052 8554331

[B307] Woo M. N. Jeon S. M. Kim H. J. Lee M. K. Shin S. K. Shin Y. C. (2010). Fucoxanthin Supplementation Improves Plasma and Hepatic Lipid Metabolism and Blood Glucose Concentration in High-Fat Fed C57BL/6N Mice. Chem. Biol. Interact. 186 (3), 316–322. 10.1016/j.cbi.2010.05.006 20519145

[B308] Woo M. N. Jeon S. M. Shin Y. C. Lee M. K. Kang M. A. Choi M. S. (2009). Anti-obese Property of Fucoxanthin Is Partly Mediated by Altering Lipid-Regulating Enzymes and Uncoupling Proteins of Visceral Adipose Tissue in Mice. Mol. Nutr. Food Res. 53 (12), 1603–1611. 10.1002/mnfr.200900079 19842104

[B309] World Health Organization (2021). Cancer. Available at: https://www.who.int/news-room/fact-sheets/detail/cancer (Accessed June 27, 2021).

[B310] World Health Organization (1981). “Toxicological Evaluation of Certain Food Additives,” in Food Additives Series (FAO/WHO) (Geneva, Switzerland: World Health Organization).

[B311] Wu M. T. Chou H. N. Huang C. J. (2014). Dietary Fucoxanthin Increases Metabolic Rate and Upregulated mRNA Expressions of the PGC-1alpha Network, Mitochondrial Biogenesis and Fusion Genes in White Adipose Tissues of Mice. Mar. Drugs 12 (2), 964–982. 10.3390/md12020964 24534841 PMC3944525

[B312] Wu M. T. Su H. M. Cui Y. Windust A. Chou H. N. Huang C. J. (2015). Fucoxanthin Enhances Chain Elongation and Desaturation of Alpha-Linolenic Acid in HepG2 Cells. Lipids 50 (10), 945–953. 10.1007/s11745-015-4059-z 26271617

[B313] Wysowski D. K. (2009). Reports of Esophageal Cancer with Oral Bisphosphonate Use. N. Engl. J. Med. 360 (1), 89–90. 10.1056/NEJMc0808738 19118315

[B314] Xia S. Wang K. Wan L. Li A. Hu Q. Zhang C. (2013). Production, Characterization, and Antioxidant Activity of Fucoxanthin from the Marine Diatom Odontella Aurita. Mar. Drugs 11 (7), 2667–2681. 10.3390/md11072667 23880936 PMC3736445

[B315] Xiangyong Y. Yixiang L. Yongpei W. Zhenhua L. Caihua G. (2014). Optimizing the Processes of Extracting and Purifying Fucoxanthin from Laminaria Japonica. J. Chin. Inst. Food Sci. Technol. 14 (3), 115–121.

[B316] Xiao H. Zhao J. Fang C. Cao Q. Xing M. Li X. (2020). Advances in Studies on the Pharmacological Activities of Fucoxanthin. Mar. Drugs 18 (12), 634. 10.3390/md18120634 PMC7763821 33322296

[B317] Yadav A. Kumari R. Yadav A. Mishra J. Srivatva S. Prabha S. (2016). Antioxidants and its Functions in Human Body-A Review. Res. Environ. Life Sci. 9 (11), 1328–1331.

[B318] Yamamoto K. Ishikawa C. Katano H. Yasumoto T. Mori N. (2011). Fucoxanthin and its Deacetylated Product, Fucoxanthinol, Induce Apoptosis of Primary Effusion Lymphomas. Cancer Lett. 300 (2), 225–234. 10.1016/j.canlet.2010.10.016 21078541

[B319] Yan X. Chuda Y. Suzuki M. Nagata T. (1999). Fucoxanthin as the Major Antioxidant in Hijikia Fusiformis, a Common Edible Seaweed. Biosci. Biotechnol. Biochem. 63 (3), 605–607. 10.1271/bbb.63.605 10227153

[B320] Yang K. M. Jeon B. H. Kim H. G. Kim J. H. (2021). Feeding Behaviors of a Sea Urchin, Mesocentrotus Nudus, on Six Common Seaweeds from the East Coast of Korea. Algae 36 (1), 51–60. 10.4490/algae.2021.36.3.5

[B321] Yang Y. P. Tong Q. Y. Zheng S. H. Zhou M. D. Zeng Y. M. Zhou T. T. (2020). Anti-inflammatory Effect of Fucoxanthin on Dextran Sulfate Sodium-Induced Colitis in Mice. Nat. Prod. Res. 34 (12), 1791–1795. 10.1080/14786419.2018.1528593 30488724

[B322] Ye J. (2013). Mechanisms of Insulin Resistance in Obesity. Front. Med. 7 (1), 14–24. 10.1007/s11684-013-0262-6 23471659 PMC3936017

[B323] Yissachar N. Zhou Y. Ung L. Lai N. Y. Mohan J. F. Ehrlicher A. (2017). An Intestinal Organ Culture System Uncovers a Role for the Nervous System in Microbe-Immune Crosstalk. Cell 168 (6), 1135–e12. 10.1016/j.cell.2017.02.009 28262351 PMC5396461

[B324] Yonekura L. Nagao A. (2007). Intestinal Absorption of Dietary Carotenoids. Mol. Nutr. Food Res. 51 (1), 107–115. 10.1002/mnfr.200600145 17195263

[B325] Yonekura L. Nagao A. (2009). Soluble Fibers Inhibit Carotenoid Micellization In Vitro and Uptake by Caco-2 Cells. Biosci. Biotechnol. Biochem. 73 (1), 196–199. 10.1271/bbb.80510 19129646

[B326] Yoshiko S. Hoyoku N. (2007). Fucoxanthin, a Natural Carotenoid, Induces G1 Arrest and GADD45 Gene Expression in Human Cancer Cells. Vivo 21 (2), 305–309. 17436581

[B327] Young I. S. Woodside J. V. (2001). Antioxidants in Health and Disease. J. Clin. Pathol. 54 (3), 176–186. 10.1136/jcp.54.3.176 11253127 PMC1731363

[B328] Yu R. X. Hu X. M. Xu S. Q. Jiang Z. J. Yang W. (2011). Effects of Fucoxanthin on Proliferation and Apoptosis in Human Gastric Adenocarcinoma MGC-803 Cells via JAK/STAT Signal Pathway. Eur. J. Pharmacol. 657 (1-3), 10–19. 10.1016/j.ejphar.2010.12.006 21187083

[B329] Yu R. X. Yu R. T. Liu Z. (2018). Inhibition of Two Gastric Cancer Cell Lines Induced by Fucoxanthin Involves Downregulation of Mcl-1 and STAT3. Hum. Cell 31 (1), 50–63. 10.1007/s13577-017-0188-4 29110251

[B330] Yuan G. Wahlqvist M. L. He G. Yang M. Li D. (2006). Natural Products and Anti-inflammatory Activity. Asia Pac J. Clin. Nutr. 15 (2), 143–152. 16672197

[B331] Zaragozá M. C. López D. Sáiz M. P. Poquet M. Pérez J. Puig-Parellada P. (2008). Toxicity and Antioxidant Activity In Vitro and In Vivo of Two Fucus Vesiculosus Extracts. J. Agric. Food Chem. 56 (17), 7773–7780. 10.1021/jf8007053 18683949

[B332] Zarros A. J. (2009). In Which Cases Is Neuroprotection Useful. Neuroprotection 1, 3–5.

[B333] Zeng H. Ishaq S. L. Zhao F. Q. Wright A. G. (2016). Colonic Inflammation Accompanies an Increase of β-catenin Signaling and Lachnospiraceae/Streptococcaceae Bacteria in the Hind Gut of High-Fat Diet-Fed Mice. J. Nutr. Biochem. 35, 30–36. 10.1016/j.jnutbio.2016.05.015 27362974

[B334] Zhang C. Zhang M. Wang S. Han R. Cao Y. Hua W. (2010). Interactions between Gut Microbiota, Host Genetics and Diet Relevant to Development of Metabolic Syndromes in Mice. ISME J. 4 (2), 232–241. 10.1038/ismej.2009.112 19865183

[B335] Zhang R. Xu M. Wang Y. Xie F. Zhang G. Qin X. (2017). Nrf2-a Promising Therapeutic Target for Defensing against Oxidative Stress in Stroke. Mol. Neurobiol. 54 (8), 6006–6017. 10.1007/s12035-016-0111-0 27696223

[B336] Zhang Y. B. Zhong Z. M. Hou G. Jiang H. Chen J. T. (2011). Involvement of Oxidative Stress in Age-Related Bone Loss. J. Surg. Res. 169 (1), e37–42. 10.1016/j.jss.2011.02.033 21529826

[B337] Zhang Z. Zhang P. Hamada M. Takahashi S. Xing G. Liu J. (2008). Potential Chemoprevention Effect of Dietary Fucoxanthin on Urinary Bladder Cancer EJ-1 Cell Line. Oncol. Rep. 20 (5), 1099–1103. 18949407

[B338] Zhao D. Kim S. M. Pan C. H. Chung D. (2014). Effects of Heating, Aerial Exposure and Illumination on Stability of Fucoxanthin in Canola Oil. Food Chem. 145, 505–513. 10.1016/j.foodchem.2013.08.045 24128507

[B339] Zhao D. Yu D. Kim M. Gu M. Y. Kim S. M. Pan C. H. (2019). Effects of Temperature, Light, and pH on the Stability of Fucoxanthin in an Oil-In-Water Emulsion. Food Chem. 291, 87–93. 10.1016/j.foodchem.2019.04.002 31006475

[B340] Zhao D. Kwon S. H. Chun Y. S. Gu M. Y. Yang H. O. (2017). Anti-Neuroinflammatory Effects of Fucoxanthin via Inhibition of Akt/NF-Κb and MAPKs/AP-1 Pathways and Activation of PKA/CREB Pathway in Lipopolysaccharide-Activated BV-2 Microglial Cells. Neurochem. Res. 42 (2), 667–677. 10.1007/s11064-016-2123-6 27933547

[B341] Zhao L. Zhang Q. Ma W. Tian F. Shen H. Zhou M. (2017). A Combination of Quercetin and Resveratrol Reduces Obesity in High-Fat Diet-Fed Rats by Modulation of Gut Microbiota. Food Funct. 8 (12), 4644–4656. 10.1039/c7fo01383c 29152632

[B342] Zheng J. Piao M. J. Kim K. C. Yao C. W. Cha J. W. Hyun J. W. (2014). Fucoxanthin Enhances the Level of Reduced Glutathione via the Nrf2-Mediated Pathway in Human Keratinocytes. Mar. Drugs 12 (7), 4214–4230. 10.3390/md12074214 25028796 PMC4113824

[B343] Zheng J. Tian X. Zhang W. Zheng P. Huang F. Ding G. (2019). Protective Effects of Fucoxanthin against Alcoholic Liver Injury by Activation of Nrf2-Mediated Antioxidant Defense and Inhibition of TLR4-Mediated Inflammation. Mar. Drugs 17 (10), 552. 10.3390/md17100552 PMC6836049 31569771

[B344] Zhou L. Ni Z. Yu J. Cheng W. Cai Z. Yu C. (2020). Correlation between Fecal Metabolomics and Gut Microbiota in Obesity and Polycystic Ovary Syndrome. Front. Endocrinol. 11. 10.3389/fendo.2020.00628 PMC7505924 33013704

[B345] Zhu Y. Cheng J. Min Z. Yin T. Zhang R. Zhang W. (2018). Effects of Fucoxanthin on Autophagy and Apoptosis in SGC-7901cells and the Mechanism. J. Cell Biochem. 119 (9), 7274–7284. 10.1002/jcb.27022 29761894

[B346] Zorofchian Moghadamtousi S. Karimian H. Khanabdali R. Razavi M. Firoozinia M. Zandi K. (2014). Anticancer and Antitumor Potential of Fucoidan and Fucoxanthin, Two Main Metabolites Isolated from Brown Algae. ScientificWorldJournal 2014, 768323. 10.1155/2014/768323 24526922 PMC3910333

